# The pluripotency factor *Nanog* regulates pericentromeric heterochromatin organization in mouse embryonic stem cells

**DOI:** 10.1101/gad.275685.115

**Published:** 2016-05-01

**Authors:** Clara Lopes Novo, Calvin Tang, Kashif Ahmed, Ugljesa Djuric, Eden Fussner, Nicholas P. Mullin, Natasha P. Morgan, Jasvinder Hayre, Arnold R. Sienerth, Sarah Elderkin, Ryuichi Nishinakamura, Ian Chambers, James Ellis, David P. Bazett-Jones, Peter J. Rugg-Gunn

**Affiliations:** 1 Epigenetics Programme, The Babraham Institute, Cambridge CB22 3AT, United Kingdom;; 2 Program in Genetics and Genome Biology, Hospital for Sick Children, Toronto, Ontario MSG 1L7, Canada;; 3 Department of Biochemistry, University of Toronto, Toronto, Ontario M5S 1A8, Canada;; 4 Program in Developmental and Stem Cell Biology, Hospital for Sick Children, Toronto, Ontario M5G 1L7, Canada;; 5 Department of Molecular Genetics, University of Toronto, Toronto, Ontario M5S 1A8, Canada;; 6 MRC Centre for Regenerative Medicine, Institute for Stem Cell Research, School of Biological Sciences, University of Edinburgh, Edinburgh EH16 4UU, United Kingdom;; 7 Nuclear Dynamics Programme, The Babraham Institute, Cambridge, CB22 3AT, United Kingdom;; 8 Department of Kidney Development, Institute of Molecular Embryology and Genetics, Kumamoto University, Kumamoto 860-0811, Japan;; 9 Centre for Trophoblast Research, University of Cambridge, Cambridge CB2 3EG, United Kingdom;; 10 Wellcome Trust-Medical Research Council Cambridge Stem Cell Institute, University of Cambridge, Cambridge CB2 1QR, United Kingdom

**Keywords:** embryonic stem cells, pluripotency, heterochromatin, nuclear organization

## Abstract

An open and decondensed chromatin organization is a defining property of pluripotency. Several epigenetic regulators have been implicated in maintaining an open chromatin organization, but how these processes are connected to the pluripotency network is unknown. Here, we identified a new role for the transcription factor NANOG as a key regulator connecting the pluripotency network with constitutive heterochromatin organization in mouse embryonic stem cells. Deletion of *Nanog* leads to chromatin compaction and the remodeling of heterochromatin domains. Forced expression of NANOG in epiblast stem cells is sufficient to decompact chromatin. NANOG associates with satellite repeats within heterochromatin domains, contributing to an architecture characterized by highly dispersed chromatin fibers, low levels of H3K9me3, and high major satellite transcription, and the strong transactivation domain of NANOG is required for this organization. The heterochromatin-associated protein SALL1 is a direct cofactor for NANOG, and loss of *Sall1* recapitulates the *Nanog*-null phenotype, but the loss of *Sall1* can be circumvented through direct recruitment of the NANOG transactivation domain to major satellites. These results establish a direct connection between the pluripotency network and chromatin organization and emphasize that maintaining an open heterochromatin architecture is a highly regulated process in embryonic stem cells.

## Abstract (precis)

Here, Novo et al. identify a new critical role for the transcription factor Nanog in maintaining an open heterochromatin state in pluripotent mouse embryonic stem cells and demonstrate that forced expression of Nanog is sufficient to remodel and decondense chromatin in more developmentally advanced mammalian cell types. This study delineates a direct connection between the pluripotency network and chromatin organization and shows that maintainence of an open heterochromatin architecture is highly regulated in embryonic stem cells.

The genome of eukaryotic cells is organized into euchromatin, which is generally permissive for gene transcription and activation, and heterochromatin, which is largely gene-poor. This form of nuclear compartmentalization is thought to impact genome regulation and stability, thereby contributing to cell identity (Fraser and Bickmore 2007; Misteli 2007; Bickmore and van Steensel 2013). Pluripotent mouse embryonic stem cell (ESC) chromatin exists in an unusual configuration with widely dispersed open chromatin throughout the nucleoplasm, including within constitutive heterochromatin domains such as pericentromeric satellite repeats (Meshorer et al. 2006; Efroni et al. 2008; Fussner et al. 2011; de Wit et al. 2013). A similar form of highly dispersed chromatin architecture also characterizes pluripotent epiblast cells within the mouse blastocyst (Ahmed et al. 2010; Boskovic et al. 2014). Upon cell differentiation, there is extensive nuclear reorganization that is associated with chromatin compaction and the formation of condensed heterochromatin domains that form a repressive environment (Meshorer et al. 2006; Efroni et al. 2008; Wen et al. 2009; Ahmed et al. 2010; Wijchers et al. 2015). Therefore, remodeling of heterochromatin architecture during stem cell and developmental fate transitions can provide an important model for investigating chromatin domain organization.

An open chromatin structure may contribute to cell pluripotency, potentially by creating a transcriptionally permissive and accessible genome (Gaspar-Maia et al. 2011; Cavalli and Misteli 2013). Reducing the expression of several epigenetic regulators (such as *Chd1*, members of the esBAF complex, and *Padi4*) in ESCs results in the accumulation of compact heterochromatin domains, disrupted self-renewal, and altered ESC differentiation potential (Meshorer et al. 2006; Gaspar-Maia et al. 2009; Lessard and Crabtree 2010; Christophorou et al. 2014). Furthermore, forced heterochromatin decompaction using DNA methyltransferase and histone deacetylase inhibitors or genetic depletion of histone H3 Lys9 methyltransferases increases the efficiency with which somatic cells can be reprogrammed to a pluripotent state (Huangfu et al. 2008; Mikkelsen et al. 2008; Soufi et al. 2012; Sridharan et al. 2013). These findings have led to the conclusion that heterochromatin regions act as impediments to the reprogramming processes and may restrict the establishment and/or maintenance of pluripotency.

In addition to influencing genome plasticity, heterochromatin organization could also have unexplored and important functions in regulating other aspects of genome function and stability in pluripotent cells. The chromatin environment of constitutive pericentromeric heterochromatin (PCH) has been well characterized in somatic cells and shown to contain condensed chromatin fibers and high levels of histone H3 Lys9 trimethylation (H3K9me3) that is mediated by *Suv39h1/2* methyltransferases (Peters et al. 2001; Lehnertz et al. 2003). The major satellite DNA repeats within PCH are typically transcriptionally repressed yet remain accessible to DNA-binding factors and are responsive to transcriptional regulation (Bulut-Karslioglu et al. 2012). Deletion of epigenetic regulators (including *Suv39h1/2* and *Dicer*) in mouse somatic cells perturbs PCH identity, causes the transcriptional up-regulation of major satellite sequences, and is associated with severe chromosome missegregation phenotypes (Peters et al. 2001; Kanellopoulou et al. 2005). Interestingly, the chromatin environment of PCH in ESCs appears to be distinct, with open and decondensed chromatin fibers and lower levels of H3K9me3 compared with somatic cells (Meshorer et al. 2006; Efroni et al. 2008; Fussner et al. 2011). The key drivers of this unusual architecture remain largely unknown, in part because the repetitive nature of heterochromatin sequences makes them challenging to study. Importantly, deletion of *Suv39h1/2* and *Dicer* in ESCs can lead to increased major satellite transcription, as in somatic cells; however, the downstream response is different because the transcriptional up-regulation does not cause chromosome missegregation in ESCs (Peters et al. 2001; Kanellopoulou et al. 2005). These findings raise the possibility that ESCs can tolerate or perhaps even require a unique PCH identity and suggest the existence of key functional differences in heterochromatin regulation between pluripotent and somatic cells.

In order to better understand how an open PCH organization is established and maintained in pluripotent cells, it is essential to dissect the functional links between pluripotency networks and nuclear architecture. One key member of the stem cell pluripotency network is the transcription factor *Nanog* (Chambers et al. 2003; Mitsui et al. 2003). Despite the central position of *Nanog* within the network, *Nanog*^–/–^ ESCs and *Nanog*^–/–^-induced pluripotent stem cells are able to undergo self-renewal and are pluripotent, suggesting that *Nanog* may have additional roles in pluripotent cells outside of controlling the transcriptional network (Chambers et al. 2007; Carter et al. 2014; Schwarz et al. 2014). We reasoned that *Nanog* is a potential candidate for regulating PCH organization in ESCs because it is expressed in cells that are associated with an open PCH architecture, such as early embryo cells and germ cells (Chambers et al. 2003; Mitsui et al. 2003; Hart et al. 2004), and we and others have shown previously that *Nanog* levels inversely correlate with several indicators of heterochromatin compaction in ESCs and embryos (Ahmed et al. 2010; Fussner et al. 2011; Mattout et al. 2011). Here, we show that *Nanog* is necessary and sufficient for PCH organization in ESCs. Deletion of *Nanog* leads to compaction and reorganization of constitutive heterochromatin domains, and forced expression of NANOG in epiblast stem cells (EpiSCs) is sufficient to decondense PCH organization and redistribute constitutive heterochromatin domains. We found that NANOG associates with satellite repeats within PCH domains, contributing to an overall heterochromatin architecture in ESCs that is characterized by highly dispersed chromatin fibers, low levels of H3K9me3, and high major satellite transcription. Importantly, tethering the NANOG transactivator domain directly to major satellite DNA is sufficient to remodel PCH organization, thereby defining a direct and active role for *Nanog* in regulating heterochromatin. Through a proteomic approach, we identified the zinc finger-containing transcription factor SALL1 as a direct NANOG-interacting protein during heterochromatin remodeling. SALL1 has a prominent heterochromatin localization in ESCs (Sakaki-Yumoto et al. 2006), and SALL1–NANOG interactions have been detected in ESCs previously (Karantzali et al. 2011); however, a functional role for *Sall1* in ESC heterochromatin regulation has not been reported. Here, we show that *Sall1*, like *Nanog*, is necessary to maintain an open heterochromatin organization in ESCs and is required for NANOG to associate with PCH in order to mediate heterochromatin remodeling. Together, these results establish the first direct molecular connection between a key member of the pluripotency network and higher-order chromatin organization in pluripotent cells and lead to the conclusion that maintaining an open and dispersed PCH architecture is a highly regulated and integrated process in ESCs.

## Results

### *Nanog* is necessary for an open heterochromatin organization in ESCs

**Figure 1.**
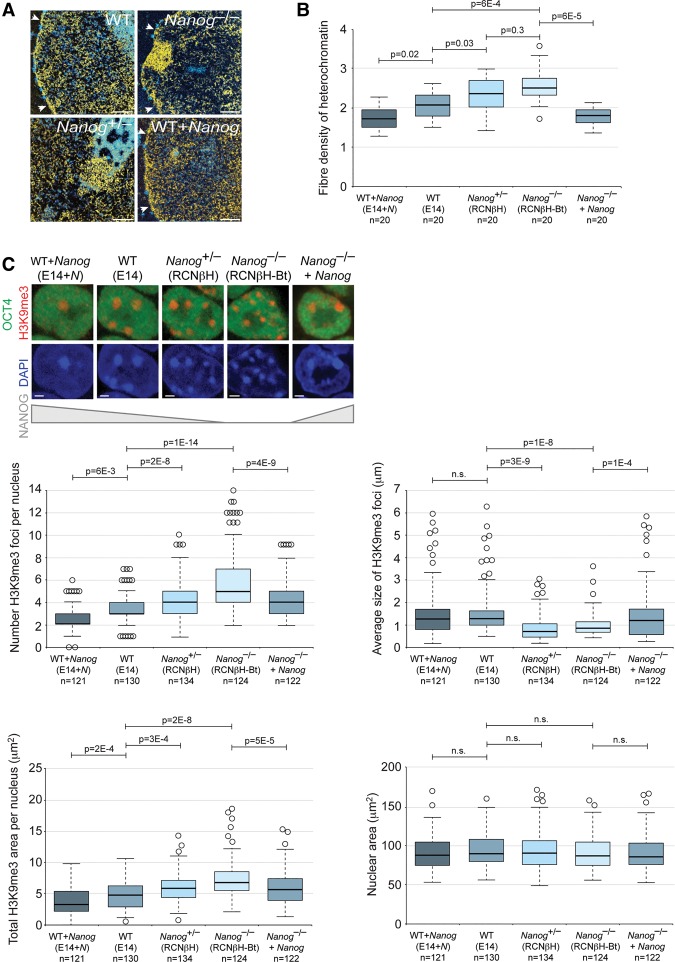
*Nanog* is required for open heterochromatin organization in ESCs. (*A*) ESI analysis of wild-type (WT), *Nanog*^–/–^, *Nanog*^+/–^, and *Nanog*-overexpressing ESCs. Quantitative phosphorus and nitrogen ratio images were segmented to show chromatin in yellow and protein-based structures in blue. The nuclear membrane is indicated with arrowheads. The regions imaged contain H3K9me3-positive PCH as determined by correlative immunofluorescent microscopy. Bar, 0.5 μm. (*B*) Box and whisker plots show the distribution of heterochromatin fiber density as revealed by phosphorus images. Data were compared using a one-way ANOVA followed by Bonferroni's multiple comparison test. (*C*) Chromocenter organization revealed by immunofluorescent analysis of H3K9me3 in ESCs expressing different levels of *Nanog*. Note that H3K9me3 foci are formed from PCH and do not overlap with other heterochromatin compartments, including telomeres. OCT4 labeling confirms the undifferentiated status of the cell type. Bar, 2 µm. Box and whisker plots show the number (*top left*), size (*top right*), and total area (*bottom left*) of H3K9me3 foci per nucleus. (*Bottom right*) Nuclear area was unchanged. Data were compared using a one-way ANOVA followed by Bonferroni's multiple comparison test. Data were collected from at least two independent experiments.

To test whether *Nanog* has a direct role in the maintenance of decondensed constitutive heterochromatin domains, we compared chromatin organization between wild-type ESCs and *Nanog*^–/–^ ESCs (Chambers et al. 2007). Electron spectroscopic imaging (ESI), a direct and quantitative technique to examine nuclear ultrastructure, confirmed that chromatin in wild-type ESCs is largely decondensed and homogenous throughout the nucleoplasm (Fig. 1A; Efroni et al. 2008). In contrast, chromatin in *Nanog*^–/–^ ESCs was less uniformly distributed, tending to compact at the nuclear envelope and nucleolar periphery into distinct heterochromatin domains (Fig. 1A). These data were supported by the increased density of heterochromatin fibers in *Nanog*^–/–^ ESCs compared with wild-type ESCs (Fig. 1B). We extended these experiments to several transgenic ESC lines representing a *Nanog* expression gradient (Chambers et al. 2007) and found a strong correlation between *Nanog* levels and heterochromatin dispersion (Fig. 1A,B).

Immunofluorescent microscopy of the heterochromatin marker H3K9me3 revealed major changes in PCH organization in *Nanog*^–/–^ ESCs. In contrast to wild-type ESCs, H3K9me3-positive chromocenters were detected as small, discrete foci in *Nanog*^–/–^ ESCs, and the median number and total area of H3K9me3-labeled foci per nucleus was significantly higher (Fig. 1C). Nuclear area was unchanged (Fig. 1C). Compared with wild-type ESCs, chromocenter number was also significantly higher in *Nanog*^+/–^ ESCs and significantly lower in *Nanog*-overexpressing ESCs, further reinforcing a correlation between *Nanog* levels and heterochromatin organization (Fig. 1C). DAPI line scan analyses demonstrated that NANOG^–/–^ ESCs chromocenters appear as distinct, bright foci and are well compartmentalized, while those of wild-type ESCs are more disrupted and dispersed with lower DAPI signal relative to nucleoplasmic background (Supplemental Fig. 1A). Differences in heterochromatin organization were confirmed using alternative wild-type and *Nanog*^–/–^ ESC lines (Supplemental Fig. 1B; Chambers et al. 2007). We also assessed whether chromocenter organization is correlated with the variegated NANOG expression that is typically observed within a colony of wild-type ESCs (Chambers et al. 2007). In agreement with our previous findings (Fussner et al. 2011), high NANOG-expressing cells exhibited larger, fewer, and more disrupted chromocenters compared with low NANOG-expressing cells (Supplemental Fig. 1C). Finally, direct visualization of PCH distribution by major satellite DNA fluorescence in situ hybridization (FISH) also revealed differences in organization between wild-type ESCs and *Nanog*^–/–^ ESCs (Supplemental Fig. 1D). Importantly, the altered heterochromatin organization observed in *Nanog*^–/–^ ESCs could be rescued by restoring NANOG levels with a transgene (Fig. 1B,C; Supplemental Fig. S1D–F).

The increased chromatin compaction and redistribution of heterochromatin domains in *Nanog*^–/–^ ESCs are similar to changes that occur upon ESC differentiation (Meshorer et al. 2006), raising the possibility that the chromatin phenotype may be caused indirectly by changes in cell state. Transcriptional and functional analyses, however, showed that *Nanog*^–/–^ ESCs retain the defining properties of wild-type ESCs. The presence of *Klf4*, *Nr0b1*, and *Zfp42* transcripts and the low level of early differentiation markers such as *T*, *Lefty1*, and *Eomes* indicate that *Nanog*^–/–^ ESCs have not initiated differentiation (Supplemental Fig. 2A), and *Nanog*^–/–^ ESCs express ESC-associated transcripts (ECATs) (Mitsui et al. 2003; Chambers et al. 2007) and known H3K9me3 methyltransferases and histone demethylases at levels similar to wild-type ESCs (Supplemental Fig. 2B). *Nanog*^–/–^ ESCs are also alkaline phosphatase-positive in a LIF-dependent manner (Chambers et al. 2007) and reveal a similar distribution of OCT4 and SOX2 protein levels within the cell population compared with wild-type ESCs (Supplemental Fig. 2C). Importantly, differences in chromocenter organization between wild-type and *Nanog*^–/–^ ESCs were retained when the analysis was restricted to KLF4-positive cells, which is a sensitive indicator of naïve pluripotency (Supplemental Fig. 2D; Guo et al. 2009), and also when cultured in more stringent 2i/LIF conditions that hold ESCs in a naïve state (Supplemental Fig. 2E; Ying et al. 2008). Together, these data show that heterochromatin compaction and redistribution occur in *Nanog*^–/–^ ESCs independently of substantial changes in cell state, thereby identifying an essential role for *Nanog* in maintaining an open heterochromatin organization in ESCs.

### Down-regulation of *Nanog* during ESC differentiation is required for heterochromatin remodeling

**Figure 2.**
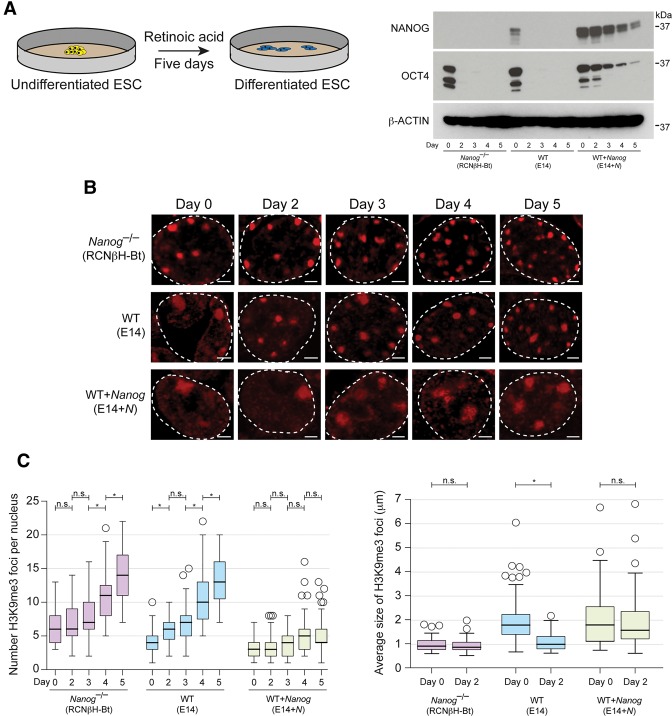
The timing of chromatin remodeling upon ESC differentiation is consistent with a role for *Nanog* in orchestrating these nuclear organization events. (*A*) Western blot of *Nanog*^–/–^, wild-type (WT), and *Nanog*-overexpressing ESCs over 5 d of differentiation with NANOG and OCT4 antibodies. (*B*) Chromocenter organization revealed by immunofluorescent analysis of H3K9me3 during ESC differentiation. (Dashed line) Nuclear periphery. Bar, 2 µm. (*C*) Box and whisker plots show the number (*left*) and size (*right*) of H3K9me3 foci per nucleus. Data were compared using a one-way ANOVA followed by Bonferroni's multiple comparison test. (n.s.) *P* > 0.1; (*) *P* < 0.01. *n* > 50 per time point.

*Nanog* is rapidly down-regulated upon ESC differentiation (Chambers et al. 2003), potentially providing a cue to condense and remodel heterochromatin architecture. To investigate whether loss of *Nanog* expression could be responsible for driving chromatin reorganization, we examined the timing of heterochromatin remodeling that occurs upon ESC differentiation. ESCs were treated with retinoic acid for 5 d, and chromocenter organization was examined every 24 h (Fig. 2A). Consistent with previous studies (Meshorer et al. 2006), PCH foci, as revealed by H3K9me3 immunofluorescent signals, became more numerous, smaller, and more intense upon ESC differentiation (Fig. 2B,C). Importantly, a major change in these parameters occurs within the first 48 h of retinoic acid induction, coinciding with loss of pluripotency factors, including NANOG (Fig. 2A,C). Therefore, the timing of heterochromatin remodeling upon the early stages of ESC differentiation is consistent with a role for *Nanog* in orchestrating these nuclear organization events.

We next assessed the impact of altering NANOG levels on chromocenter remodeling during ESC differentiation. At day 0, *Nanog*^–/–^ ESCs already displayed well-defined and discrete chromocenters, and this distribution did not significantly change over the first 3 d of ESC differentiation, suggesting that there is little PCH remodeling during this period in the absence of NANOG (Fig. 2B,C). Interestingly, a subsequent phase of chromocenter remodeling occurred after day 3, pointing to the existence of a later stage NANOG-independent process. Conversely, continuous ectopic expression of NANOG in wild-type ESCs prevented the typical remodeling in chromocenter organization, instead maintaining the highly disrupted PCH organization that is characteristic of undifferentiated ESCs (Fig. 2B,C). This phenotype could be direct or indirect because it coincided with the failure to down-regulate pluripotency factors such as OCT4, a finding that is consistent with our previously published differentiation experiments (Chambers et al. 2003). Overall, these results identify a dependency between NANOG levels and PCH remodeling during ESC differentiation.

### *Nanog* is sufficient to remodel heterochromatin state

**Figure 3.**
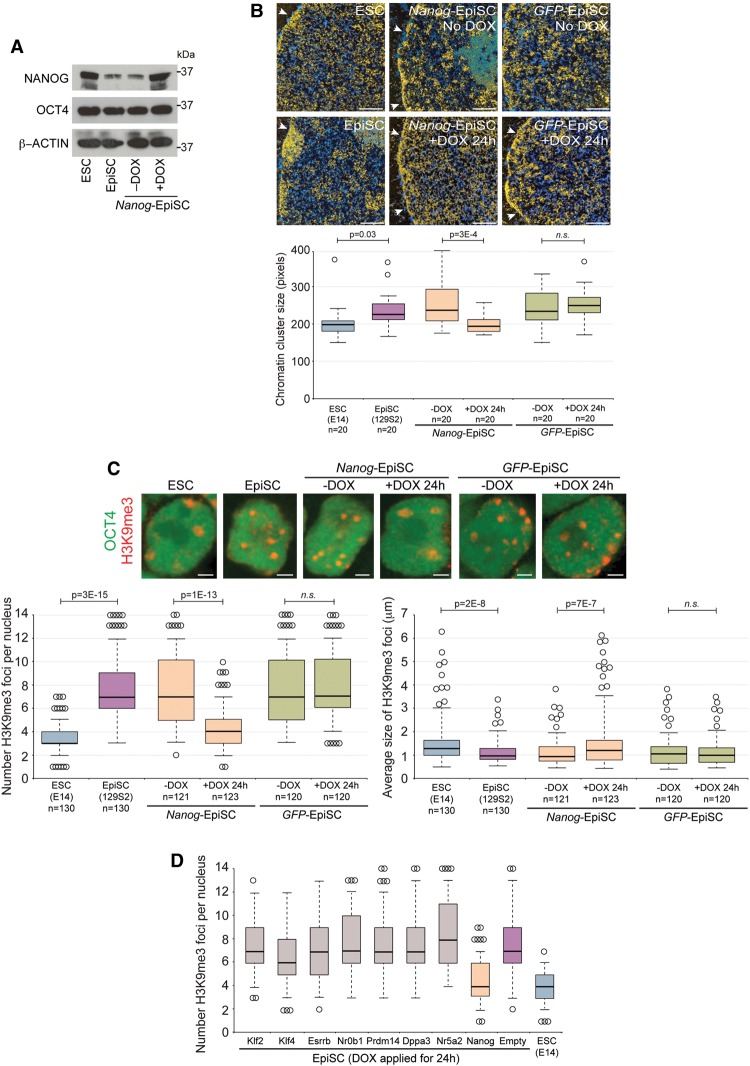
*Nanog* is sufficient to remodel heterochromatin in EpiSCs. (*A*) Western blot of NANOG and OCT4 in ESCs, EpiSCs, and doxycycline (DOX)-inducible *Nanog*-EpiSCs. DOX was applied for 24 h. (*B*) ESI analysis of ESCs, EpiSCs, DOX-inducible *Nanog*-EpiSCs, and DOX-inducible *GFP*-EpiSCs. DOX was applied for 24 h. The nuclear membrane is indicated by arrowheads. Bar, 0.5 μm. Box and whisker plots reveal the distribution in size of the chromatin clusters. *P*-values were calculated using Student's *t*-test. (*C*) Chromocenter organization revealed by immunofluorescent analysis of H3K9me3. OCT4 labeling confirmed the undifferentiated status of the cell type. DOX was applied for 24 h. Bar, 2 µm. Box and whisker plots show the number (*left*) and size (*right*) of H3K9me3 foci per nucleus. *P*-values were calculated using Student's *t*-test. (n.s.) *P* > 0.1. Data were collected from at least two independent experiments. (*D*) Several pluripotency factors were overexpressed in EpiSCs for 24 h; only *Nanog* was able to remodel chromocenter organization. Box and whisker plots show the number of H3K9me3 foci per nucleus. *n* = 50 per cell line (images are shown in Supplemental Fig. 4A.)

To further explore the impact of early differentiation events on heterochromatin organization, we examined chromatin organization in EpiSCs. EpiSCs are capable of differentiating into all three germ layers and express several pluripotency factors, such as OCT4, at levels similar to ESCs but importantly express NANOG at lower levels compared with ESCs (Fig. 3A; Supplemental Fig. 2A; Brons et al. 2007; Tesar et al. 2007; Osorno and Chambers 2011; Osorno et al. 2012). We hypothesized that if *Nanog* was instructive in maintaining a decondensed heterochromatin organization, then EpiSCs may reveal a more compacted chromatin architecture. Indeed, ultrastructural analysis using ESI showed that chromatin in EpiSCs was organized into distinct compacted chromatin domains and generally was less uniformly distributed compared with ESCs (Fig. 3B). Differences in chromatin organization between EpiSCs and ESCs was confirmed by immunofluorescent microscopy of heterochromatin foci identified by H3K9me3 and by DNA FISH for major satellites, revealing that chromocenters are organized into small discrete foci in EpiSCs (Fig. 3C; Supplemental Fig. 3A). Together, these data reveal that chromatin in EpiSCs is organized similarly to *Nanog*^–/–^ ESCs, thereby reinforcing a functional link between *Nanog* and heterochromatin organization.

As forced NANOG expression could restore typical ESC chromatin architecture in *Nanog*^–/–^ ESCs, we reasoned that elevated expression of NANOG could also be sufficient to remodel heterochromatin organization in EpiSCs. Prolonged NANOG expression has been shown previously to enable EpiSC reprogramming (Silva et al. 2009), potentially confounding analysis of chromatin remodeling. We therefore designed experiments to investigate the effects of short-term NANOG induction in EpiSCs. We engineered EpiSC lines that expressed *Nanog* upon doxycycline (DOX) treatment, thereby allowing precise control of the timing of NANOG induction (*Nanog*-EpiSCs) (Fig. 3A; Supplemental Fig. 3B). Remarkably, direct analysis of chromatin organization using ESI and major satellite DNA FISH in addition to indirect indicators, including H3K9me3 immunofluorescent signals and DAPI line scan analyses, revealed that heterochromatin was remodeled and dispersed within 24 h of NANOG induction (Fig. 3B,C; Supplemental Fig. 3C,D). At this time point, the majority of chromatin was uniformly distributed throughout the nucleoplasm, with an overall chromatin architecture indistinguishable from ESCs. As expected, control cells, including noninduced *Nanog*-EpiSCs and induced *GFP*-EpiSCs, revealed chromatin organization typical of EpiSCs, with domains of compacted chromatin and the presence of small heterochromatin foci (Fig. 3B,C; Supplemental Fig. 3C,D). Induced expression of a NANOG homeodomain point mutant that has substantially reduced DNA-binding affinity (N51E) (Jauch et al. 2008) was unable to remodel heterochromatin architecture, suggesting that a functional homeodomain is required (Supplemental Fig. 3E,F). We confirmed that *Nanog* expression driven from a constitutive promoter in EpiSCs was also sufficient to remodel heterochromatin (data not shown). Importantly, short-term forced expression of alternative pluripotency factors, including *Klf2*, *Klf4*, *Esrrb*, *Nr0b1*, *Prdm14*, *Dppa3*, and *Nr5a2*, did not cause detectable changes in heterochromatin organization, underscoring the specific role for *Nanog* in remodeling chromatin organization (Fig. 3D; Supplemental Fig. 4A,B). In addition, NANOG was unable to access and remodel heterochromatin when overexpressed in fibroblasts, indicating that the function may be restricted to early embryo or stem cell types (Supplemental Fig. 4C,D; data not shown). Last, investigation of EpiSC status upon chromatin remodeling revealed unchanged epigenetic and transcriptomic profiles after 24 h of NANOG expression (Supplemental Fig. 4E,F), thereby indicating that NANOG-induced heterochromatin reorganization occurs independently of EpiSC-to-ESC reprogramming. Collectively, these results demonstrate that NANOG is sufficient to remodel heterochromatin in EpiSCs, resulting in an open chromatin architecture that is indistinguishable from ESCs. Importantly, these remodeling events can precede changes in other epigenetic and transcriptional events, implying that heterochromatin organization can be decoupled from cell state.

### *Nanog*-dependent pericentromeric satellite organization in ESCs

**Figure 4.**
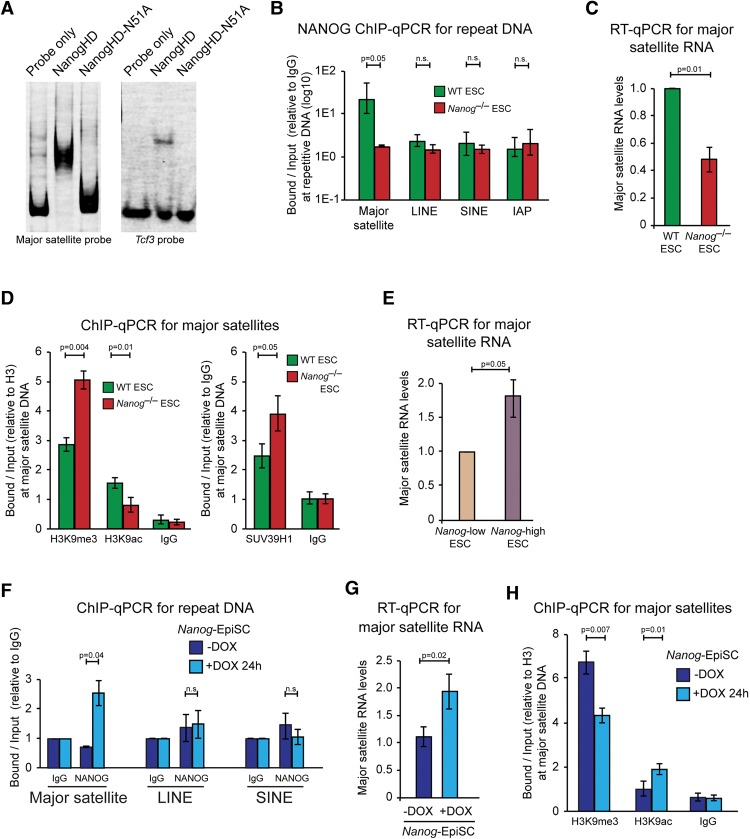
NANOG associates with major satellite repeats in ESCs. (*A*) His-tagged recombinant wild-type and N51A mutant NANOG homeodomains were used for electrophoretic mobility shift assays with a full-length major satellite probe (234 bp) (Bulut-Karslioglu et al. 2012) and a *Tcf3* probe (14 bp) (Jauch et al. 2008). (*B*) ChIP-qPCR analysis of NANOG at major satellite, LINE, SINE, and IAP DNA in wild-type and *Nanog*^–/–^ ESCs. (*C*) RT-qPCR for major satellite transcripts in wild-type and *Nanog*^–/–^ ESCs. Values were normalized to *Hmbs* and are shown relative to wild type. (*D*) ChIP-qPCR for H3K9me3, H3K9ac, and IgG (normalized to unmodified H3) (*left*) and SUV39H1 and IgG (normalized to IgG) (*right*) at major satellite DNA in wild-type and *Nanog*^–/–^ ESCs. (*E*) RT-qPCR for major satellite transcripts in ESCs that were separated by flow cytometry for *Nanog* low-expressing and *Nanog* high-expressing cells using an ESC line with *eGFP* inserted into one *Nanog* allele (TNGA). (*F*) ChIP-qPCR analysis of NANOG at major satellite DNA, LINE, and SINE in *Nanog*-EpiSCs with and without 24 h of DOX induction. (*G*) RT-qPCR for major satellite transcripts in *Nanog*-EpiSCs with and without 24 h of DOX induction. Values were normalized to *Hmbs.* (*H*) ChIP-qPCR for H3K9me3, H3K9ac, and IgG at major satellite DNA in *Nanog*-EpiSCs with and without 24 h of DOX induction. Values were normalized to unmodified H3. All data represent mean ± SD from three biological experiments.

Our DNA FISH experiments revealed that pericentromeric major satellite sequences that cluster within chromocenters undergo substantial remodeling in *Nanog*^–/–^ ESCs (Supplemental Fig. 1D). Given that transcription factors can directly control major satellite DNA in other cell types (Bulut-Karslioglu et al. 2012), we hypothesized that NANOG could regulate the chromatin state of major satellite repeats in ESCs, thereby contributing to PCH organization. To test this hypothesis, we performed electrophoretic mobility shift assays to assess direct binding of the NANOG homeodomain to major satellite repeats. We found that the recombinant NANOG homeodomain was able to substantially reduce the mobility of the full-length major satellite probe, and the shift was even more pronounced than that of a probe containing a well-characterized NANOG-binding site within the *Tcf3* promoter (Fig. 4A; Jauch et al. 2008). A point mutation in the recognition helix of the NANOG homeodomain (N51A) (Jauch et al. 2008) abolished DNA interaction with the major satellite and *Tcf3* probes (Fig. 4A), demonstrating that a functional homeodomain is required for major satellite DNA binding. We next examined NANOG occupancy at PCH in ESCs using chromatin immunoprecipitation (ChIP). ChIP experiments revealed that NANOG bound to major satellite repeats in wild-type ESCs but not to other repeat classes (Fig. 4B). The association of NANOG with major satellite repeats corresponded with several hallmarks of an open PCH organization. First, we examined RNA output using RT-qPCR with a primer pair that amplifies one unit of the 234-base-pair (bp) mouse major satellite repeat (Lehnertz et al. 2003). Major satellite transcripts were significantly decreased (approximately twofold) in *Nanog*^–/–^ ESCs compared with wild-type ESCs (Fig. 4C). Second, ChIP analyses revealed that H3K9me3 levels were approximately twofold increased and that H3K9 acetylation (H3K9ac) levels were approximately twofold decreased at major satellite repeats in *Nanog*^–/–^ ESCs compared with wild-type ESCs, and this difference was associated with increased occupancy of the H3K9 methyltransferase SUV39H1 (Fig. 4D), together indicating an accumulation of heterochromatinization at major satellite repeats in *Nanog*^–/–^ ESCs. Other repeat sequences such as LINE, SINE, and IAP were unaffected (Supplemental Fig. 5A). Immunofluorescent microscopy of NANOG localization revealed a strong pan-nuclear signal that was not enriched or depleted at chromocenters (Supplemental Fig. 5B). Last, separation of ESCs based on variegated *Nanog* levels revealed that high *Nanog*-expressing ESCs transcribed higher levels of major satellite RNA compared with low *Nanog*-expressing ESCs, further reinforcing the connection between NANOG protein levels and a more open PCH organization (Fig. 4E).

We next investigated NANOG binding and RNA output in EpiSCs upon chromatin remodeling. ChIP experiments showed increased NANOG occupancy at major satellite repeats, but not at LINE and SINE sequences, upon *Nanog* induction in EpiSCs (Fig. 4F). The binding events correlated with RNA output from major satellite repeats, which significantly increased (approximately twofold) after 24 h of *Nanog* induction in EpiSCs (Fig. 4G). At this time point, major satellite transcripts reached the same level as wild-type ESCs. Consistent with these changes, ChIP analyses revealed that H3K9me3 levels at major satellite DNA significantly decreased and that H3K9ac levels increased in *Nanog*-overexpressing EpiSCs compared with noninduced EpiSCs (Fig. 4H). Importantly, expression of a *Nanog* homeodomain point mutant that has substantially reduced DNA-binding affinity (N51E) (Jauch et al. 2008) was unable to induce changes in major satellite transcription and H3K9me3 levels in EpiSCs, demonstrating that a functional homeodomain is required (Supplemental Fig. 5C). Last, as major satellite transcription could be cell cycle-regulated (Lu and Gilbert 2007), we examined whether changes in cell cycle timing may contribute to the observed changes in RNA output from major satellite repeats. Flow cytometry analysis revealed that cell cycle parameters were unaltered in *Nanog*^–/–^ ESCs and also upon *Nanog* induction in EpiSCs (Supplemental Fig. 5D). Together, these results establish that NANOG binding is associated with increased major satellite transcription and decreased heterochromatinization of major satellite repeats, underlying the role of *Nanog* in maintaining an open PCH organization in pluripotent cells.

### The NANOG transactivation domain is critical for heterochromatin remodeling

**Figure 5.**
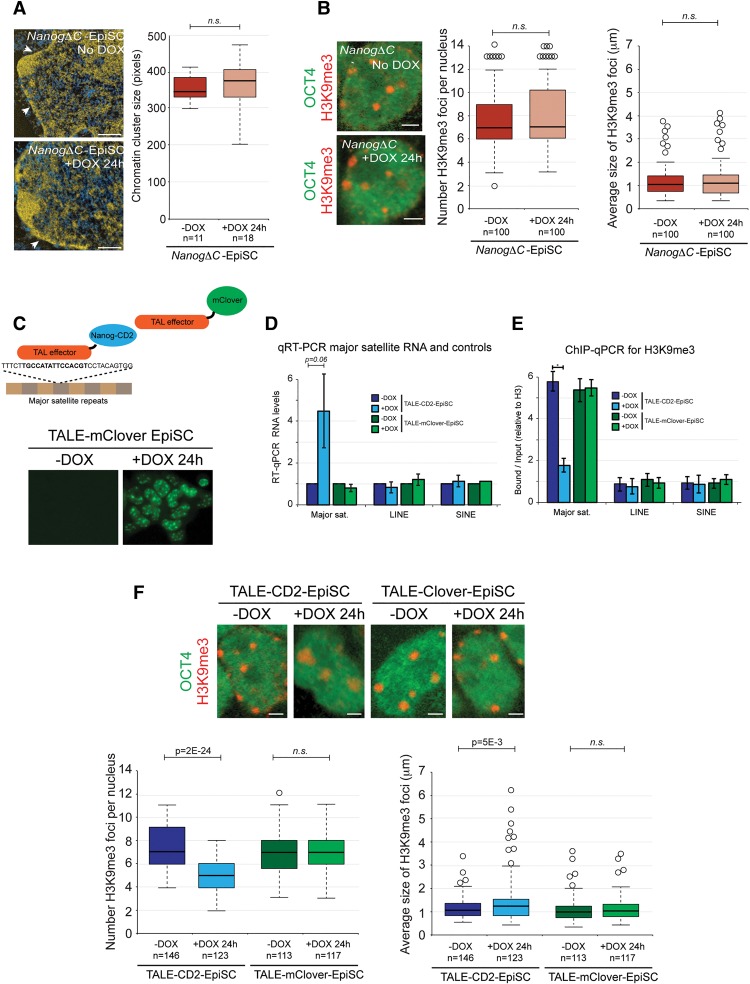
The NANOG transactivation domain is necessary and sufficient for heterochromatin remodeling. (*A*) ESI analysis of DOX-inducible *NanogΔC*-EpiSCs. DOX was applied for 24 h. The nuclear membrane is indicated by arrowheads. Bar, 0.5 μm. Box and whisker plots reveal the distribution in size of chromatin clusters. *P*-value was calculated using Student's *t*-test. (n.s.) *P* > 0.1. (*B*) Chromocenter organization revealed by immunofluorescent analysis of H3K9me3. OCT4 labeling confirmed the undifferentiated status of the cell type. DOX was applied for 24 h. Bar, 2 μm. Box and whisker plots show the number (*left*) and size (*right*) of H3K9me3 foci per nucleus. *P*-values were calculated using Student's *t*-test. (n.s.) *P* > 0.1. (*C*) Diagram of transcription activator-like effector (TALE)-CD2 and TALE-mClover fusion proteins and a fluorescent microscopy image demonstrating localization of TALE-mClover to chromocenters after 24 h of DOX induction in EpiSCs. (*D*) RT-qPCR for major satellite transcripts, LINE, and SINE in *TALE-CD2*-EpiSCs and *TALE-mClover*-EpiSCs with and without 24 h of DOX induction. Values were normalized to *Hmbs.* (*E*) ChIP-qPCR for H3K9me3 at major satellite, LINE, and SINE DNA in *TALE-CD2*-EpiSCs and *TALE-mClover*-EpiSCs with and without 24 h of DOX induction. Values were normalized to unmodified H3. All data represent mean ± SD from at least three biological experiments. (*F*) Chromocenter organization revealed by immunofluorescent analysis of H3K9me3. Box and whisker plots show the number (*left*) and size (*right*) of H3K9me3 foci per nucleus.

We next examined the molecular basis for *Nanog*-dependent PHC organization. Transactivation activity of NANOG can be mediated via the C-terminal WR and CD2 domains (Supplemental Fig. 6A; Pan and Pei 2003; Oh et al. 2005). We hypothesized that the recruitment of the transactivation domains to major satellite repeats could underlie the open PCH organization typical of ESCs. To test this hypothesis, we first expressed a *Nanog* transgene that lacked the WR and CD2 transactivation domains (*NanogΔC*) in EpiSCs (Supplemental Fig. 6A–C). ESI analysis and H3K9me3 immunofluorescent microscopy revealed that *NanogΔC* was unable to decompact chromatin, remodel chromocenter organization, or up-regulate major satellite transcription (Fig. 5A,B; Supplemental Fig. 6D). These findings demonstrate the requirement for the transactivation domain in *Nanog*-mediated heterochromatin remodeling.

To investigate whether recruitment of NANOG transactivation domains to PCH was sufficient to initiate chromatin remodeling, we constructed a fusion protein between the CD2 transactivation domain and a transcription activator-like effector (TALE) that is known to specifically bind mouse major satellite DNA (TALE-CD2) (Miyanari et al. 2013). A luciferase-based reporter assay confirmed the activity of the fusion protein (Supplemental Fig. 6E). As a control, we used a previously published and characterized TALE-mClover protein, which binds to major satellite DNA but does not alter the transcriptional or epigenetic properties of the target sequences (Supplemental Fig. 6E; Miyanari et al. 2013). We engineered EpiSC lines with DOX-inducible TALE-CD2 or TALE-mClover transgenes and confirmed that addition of DOX to the culture medium caused up-regulation of the transgenes and localization of the fusion proteins to PCH (Fig. 5C). After 24 h of DOX induction, TALE-CD2, but not TALE-mClover, caused major satellite repeats to adopt a more open and active state, as shown by a significant transcriptional up-regulation and corresponding changes in H3K9me3 and H3K9ac levels (Fig. 5D,E). Other repeat classes such as SINE and LINE were unaffected. Importantly, induction of TALE-CD2 in EpiSCs was also sufficient to remodel chromocenter organization such that chromocenters adopted a highly disrupted and dispersed pattern that is typical of undifferentiated ESCs (Fig. 5F). Of note is that induction of a substantially stronger transactivator (TALE-VP64) in EpiSCs caused a severe phenotype with irregular nuclear morphology (data not shown), suggesting that transactivator strength is important. Together, these results show that recruitment of the NANOG transactivator domain specifically to major satellite sequences is able to recapitulate the phenotype induced by overexpression of full-length *Nanog*, thereby identifying a direct and active role for *Nanog* in regulating PCH organization.

### *Sall1* is required for *Nanog*-mediated remodeling

**Figure 6.**
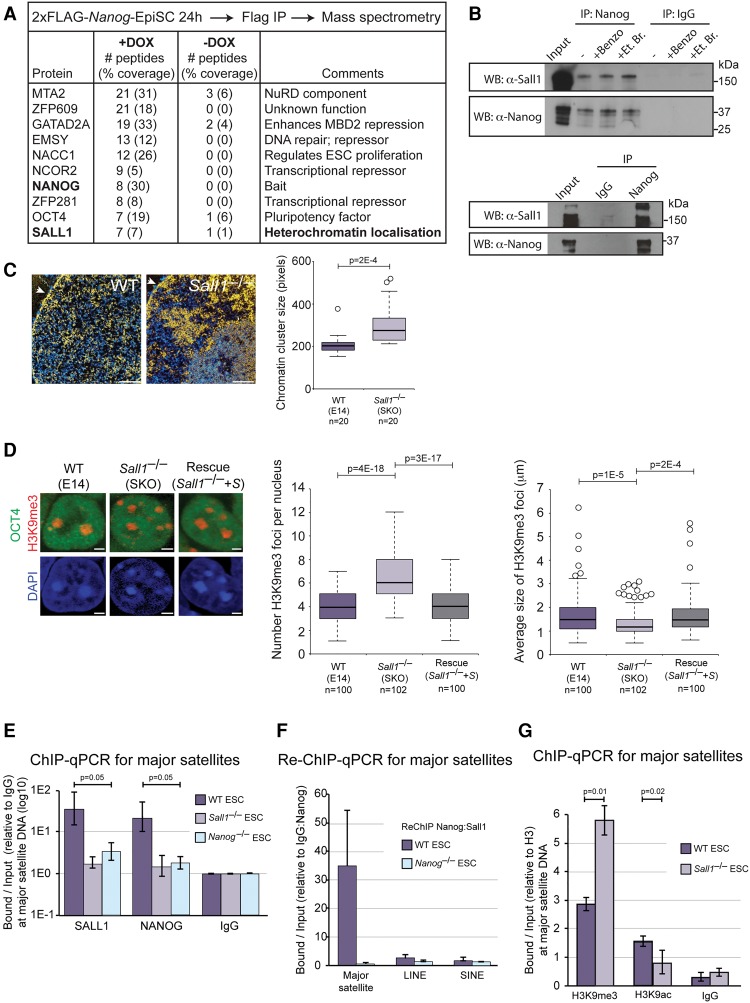
SALL1 binds NANOG directly and is required for open heterochromatin organization in ESCs. (*A*) Table showing a subset of proteins copurifying with 2xFlag-*Nanog* in EpiSCs, as identified by mass spectrometry. (*B*, *top*) Coimmunoprecipitation of endogenous NANOG from wild-type ESC nuclear extracts, analyzed by Western blot (WB). Benzonase (Benzo) and ethidium bromide (Et. Br.) were added where indicated. (*Bottom*) Coimmunoprecipitation of recombinant NANOG and SALL1, analyzed by Western blot. (*C*) ESI analysis of wild-type (WT) and *Sall1*^–/–^ ESCs. The nuclear membrane is indicated by an arrowhead. Bar, 0.5 μm. Box and whisker plots reveal the distribution in size of chromatin clusters. *P*-value was calculated using Student's *t*-test. Heterochromatin fiber density was also significantly increased in *Sall1*^–/–^ ESCs (data not shown). (*D*) Chromocenter organization revealed by immunofluorescent analysis of H3K9me3. OCT4 labeling confirmed the undifferentiated status of the cell type. Bar, 2 µm. Box and whisker plots show the number (*left*) and size (*right*) of H3K9me3 foci per nucleus. Data were compared using a one-way ANOVA followed by Bonferroni's multiple comparison test. Data were collected from at least two independent experiments. (*E*) ChIP-qPCR analysis of SALL1 and NANOG at major satellite DNA in wild-type, *Sall1*^–/–^, and *Nanog*^–/–^ ESCs. (*F*) Re-ChIP-qPCR analysis of NANOG and SALL1 co-occupancy at major satellite, LINE, and SINE DNA in wild-type and *Nanog*^–/–^ ESCs. (*G*) ChIP-qPCR for H3K9me3, H3K9ac, and IgG at major satellite DNA in wild-type and *Sall1*^–/–^ ESCs. Values were normalized to unmodified H3. All qPCR data represent mean ± SD from three biological experiments.

To uncover the molecular mechanisms through which NANOG can associate with PCH in order to actively regulate major satellite repeats, we identified proteins that interact with NANOG during PCH remodeling. We generated EpiSCs containing a DOX-inducible 2xFlag-*Nanog* transgene (Supplemental Fig. 6B,C). The tagged protein was functional, as it was able to remodel heterochromatin when overexpressed in EpiSCs, and was sufficient to enable LIF-independent ESC proliferation (data not shown). We expressed the transgene for 24 h in EpiSCs, immunopurified Flag-containing protein complexes, and identified associated proteins by mass spectrometry. As a control, we examined the same EpiSC line without DOX induction. Out of the proteins detected, we focused on those previously shown to interact with NANOG in ESCs (Wang et al. 2006; Costa et al. 2013; Gagliardi et al. 2013), as they are the most likely candidates for establishing and maintaining heterochromatin identity in pluripotent cells (Fig. 6A). In particular, the interaction partner SALL1 was of interest because it has been shown previously to bind heterochromatin domains in ESCs (Sakaki-Yumoto et al. 2006) and could therefore provide a link between NANOG and recruitment to PCH. We used endogenous coimmunoprecipitation to confirm the association in 2xFlag-*Nanog*-EpiSCs (data not shown) and wild-type ESCs (Fig. 6B) as well as the direct interaction of recombinant NANOG and SALL1 proteins (Fig. 6B). We also verified that SALL1 is present at similar levels in ESCs and EpiSCs (Supplemental Fig. 7A).

Despite the prominent heterochromatin localization of SALL1 in ESCs (Sakaki-Yumoto et al. 2006), a functional role for *Sall1* in heterochromatin regulation has not been reported. We therefore examined chromatin organization in *Sall1*^–/–^ ESCs (Yuri et al. 2009) in order to establish whether *Sall1*, like *Nanog*, is required to maintain open heterochromatin. Ultrastructural analysis using ESI revealed that chromatin in *Sall1*^–/–^ ESCs was highly heterogeneous, frequently forming regions of compact chromatin at the nuclear envelope and nucleolar periphery (Fig. 6C). Chromatin cluster size and heterochromatin fiber density were significantly higher in *Sall1*^–/–^ ESCs compared with wild-type ESCs (Fig. 6C). The altered chromatin architecture in *Sall1*^–/–^ ESCs was confirmed by H3K9me3 and DAPI line scan immunofluorescence microscopy as well as major satellite DNA FISH (Fig. 6D; Supplemental Fig. 7B,C) and also when cultured in 2i/LIF conditions (Supplemental Fig. 7D). The alteration in chromatin organization observed in *Sall1*^–/–^ ESCs could be rescued by restoring *Sall1* levels with a transgene (Fig. 6D; Supplemental Fig. 7C,E,F). Therefore, the inactivation of *Sall1* phenocopies the defects in PCH organization observed in *Nanog*^–/–^ ESCs. Importantly, NANOG levels are unchanged in *Sall1*^–/–^ ESCs and therefore remain highly expressed, and SALL1 levels are unchanged in *Nanog*^–/–^ ESCs and *Nanog*-overexpressing EpiSCs (Supplemental Figs. 1F, 6B, 7E). Furthermore, transcripts that characterize wild-type ESCs are unaltered in *Sall1*^–/–^ ESCs, demonstrating that loss of *Sall1* perturbs chromatin organization without alteration of ESCs’ identities (Supplemental Fig. 7G,H).

At the molecular level, ChIP analysis confirmed that SALL1 binds to major satellite DNA in wild-type ESCs (Fig. 6E). Moreover, re-ChIP demonstrated that NANOG and SALL1 co-occupy major satellite DNA in wild-type ESCs, as expected given their direct interaction (Fig. 6F). Importantly, deletion of *Sall1* in ESCs leads to loss of NANOG binding to major satellite DNA, thereby demonstrating a requirement for SALL1 in enabling NANOG occupancy at PCH repeats (Fig. 6E). SALL1 binding was also reduced at major satellite DNA in *Nanog*^–/–^ ESCs (Fig. 6E). Deletion of *Sall1* leads to a reorganization of the PCH state that is characterized by increased levels of H3K9me3, decreased levels of H3K9ac, and down-regulated major satellite transcription (Fig. 6G; Supplemental Fig. 7I). Other repeat sequences such as LINE, SINE, and IAP were unaffected (Supplemental Fig. 7J). Together, these findings identify a role for SALL1 in regulating PCH organization in ESCs.

**Figure 7.**
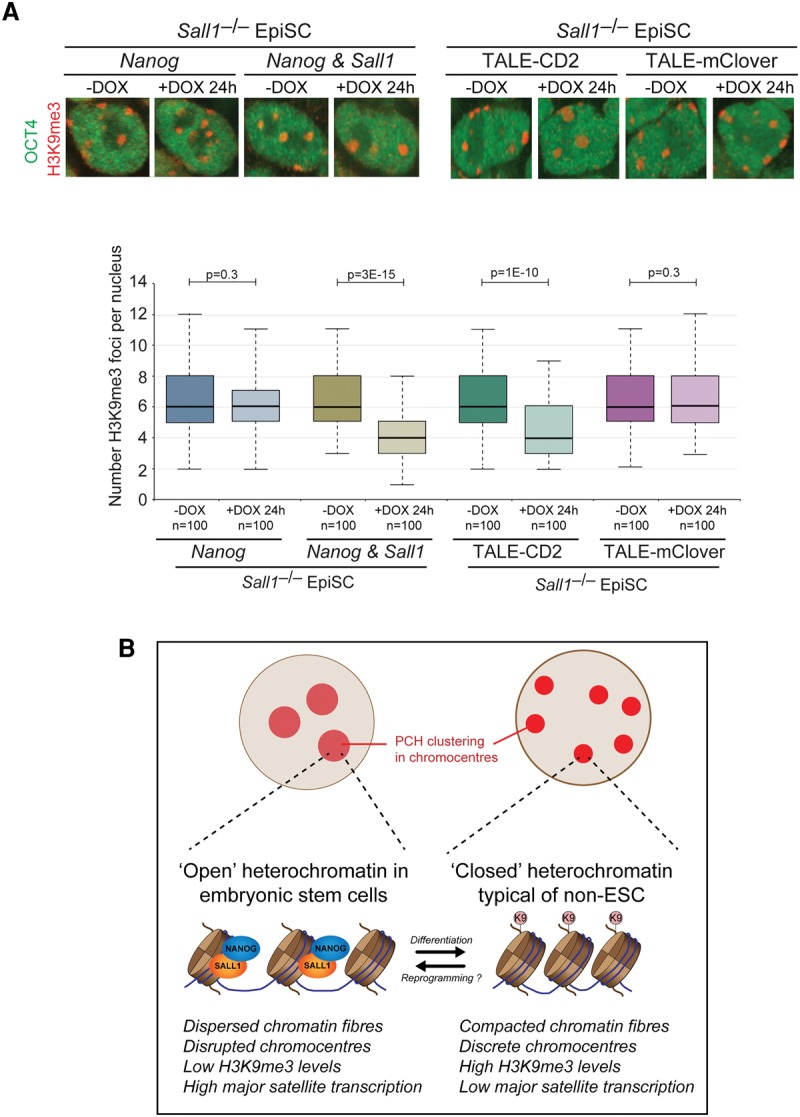
*Sall1* is required for *Nanog*-mediated heterochromatin remodeling. (*A*) *Nanog* is unable to remodel chromocenter organization in the absence of *Sall1*, but recruitment of NANOG-CD2 directly to major satellites can bypass the requirement for *Sall1*. Chromocenter organization revealed by immunofluorescent analysis of H3K9me3. OCT4 labeling confirmed the undifferentiated status of the cell type. DOX was applied for 24 h. Bar, 2 µm. Box and whisker plots show the number of H3K9me3 foci per nucleus. *P*-values were calculated using Student's *t*-test. Data were collected from at least two independent experiments. (*B*) Model illustrating the role of *Nanog* in maintaining an open heterochromatin organization in pluripotent cells.

Based on the above results, we propose that NANOG and SALL1 codependently maintain heterochromatin organization in ESCs. To further test this model, we next addressed whether SALL1 is required for NANOG-mediated heterochromatin reorganization in EpiSCs. We generated *Sall1*^–/–^ EpiSCs that contained a DOX-inducible *Nanog* transgene (Supplemental Fig. 7K). After NANOG induction in these cells, there was no difference in the number and appearance of chromocenters (Fig. 7A). In contrast, NANOG induction together with restoration of SALL1 levels resulted in chromocenter reorganization to levels typical of ESCs (Fig. 7A), demonstrating that SALL1 is a necessary cofactor for NANOG-mediated heterochromatin remodeling. Importantly, the requirement for SALL1 in heterochromatin remodeling could be bypassed through expression of TALE-CD2, which was able to remodel chromocenter organization in *Sall1*^–/–^ EpiSCs (Fig. 7A). We therefore propose a model in which SALL1 is required for NANOG binding at major satellite pericentromeric repeats (Fig. 7B). Once at the repeats, the strong transactivation domains of NANOG are able to promote a more open and active chromatin state at PHC domains.

## Discussion

An open and highly dispersed chromatin architecture is a defining property of naïve pluripotency in vitro and in vivo (Meshorer et al. 2006; Efroni et al. 2008; Ahmed et al. 2010; Boskovic et al. 2014). We identified a critical new role for the transcription factor *Nanog* in the establishment and maintenance of open heterochromatin, thereby forming a direct link between the ESC regulatory network and nuclear organization in pluripotent cells. Characterization of the mechanism revealed a requirement for the C-terminal transactivation domains of NANOG to be recruited to PCH and the associated regulation of satellite repeats. An alternative set of transcription factors has been shown to maintain heterochromatin state through satellite regulation in fibroblast cells (Bulut-Karslioglu et al. 2012), an indication that this mode of chromatin regulation may be common but involves cell type-specific transcription factors. In ESCs, *Nanog* is likely to function together with key chromatin regulators, such as *Chd1*, to orchestrate an open higher-order chromatin structure (Gaspar-Maia et al. 2011). Our analysis of NANOG-interacting proteins now provides a set of additional factors that may have functional roles in controlling chromatin organization in ESCs and will be a focus of future research.

Constitutive heterochromatin is rapidly compacted upon ESC differentiation and embryo development, implying close coordination of chromatin with cell status (Meshorer et al. 2006; Ahmed et al. 2010; Boskovic et al. 2014). Our findings suggest that down-regulation of *Nanog*, one of the earliest events in ESC differentiation, is a key driver of heterochromatin compaction. Conversely, heterochromatin decompaction and *Nanog* induction are critical events that co-occur at a late stage in cell reprogramming (Brambrink et al. 2008; Silva et al. 2009; Fussner et al. 2011; Schwarz et al. 2014). Therefore, our findings also have important consequences for controlling heterochromatin organization during reprogramming, an event that has been shown previously to be a barrier to reprogramming efficiency (Soufi et al. 2012). The precise timing of the molecular events that lead to heterochromatin remodeling during reprogramming will be important to investigate further (Mattout et al. 2011).

Given that PCH organization is a highly regulated process in ESCs and, as we show here, has been integrated within the pluripotency network, what could be the role of an open chromatin architecture in ESCs? So far, the prevailing model to explain the function of an open chromatin configuration in ESCs proposes that it helps maintain genome plasticity (Gaspar-Maia et al. 2011; Cavalli and Misteli 2013). Our results reinforce the concept that the chromatin state of PCH domains is maintained in an unusually open and active form. Interestingly, a recent study identified genomic regions that loop and physically interact with PCH (Wijchers et al. 2015). Transcriptional and epigenetic approaches demonstrated that PCH domains are not a strong repressive environment in ESCs, but, instead, this property is acquired upon ESC differentiation (Wijchers et al. 2015). These findings are consistent with a corresponding accumulation of repressive marks at PCH domains upon ESC differentiation and in somatic cells. It is therefore possible that PCH domains are organized and controlled in ESCs to prevent the unwanted strong repression that could potentially restrict genome regulation. Importantly, our results show that *Nanog*^–/–^ ESCs can tolerate compaction of heterochromatin domains without substantial changes in cell state or the ability of the cells to differentiate into all three germ layers (Chambers et al. 2007). However, *Nanog*^–/–^ ESCs are compromised, as they do exhibit diminished colony formation and are more prone to spontaneous differentiation than wild-type ESCs (Mitsui et al. 2003; Chambers et al. 2007). In addition, *Sall1*-deficient embryos and ESCs have no apparent defects in pluripotency or early development (Nishinakamura et al. 2001; Yuri et al. 2009) despite the demonstration here that *Sall1* is required for open heterochromatin organization in ESCs. Therefore, it is possible that compaction of heterochromatin domains may destabilize and restrict ESCs but that additional events are required to trigger functional changes in ESC state. The same model may be true of pluripotent cells in vivo and thereby account for the absence of an early developmental phenotype in *Sall1* mutant embryos, although the regulative nature of early development may also result in compromised or unfit cells being excluded from the embryo.

An open and active PCH configuration with relatively low levels of heterochromatin modifications could be functionally linked with the observation that pericentromeric-associated proteins bind more loosely or are absent in ESCs (Meshorer et al. 2006; Melcer et al. 2012; Mattout et al. 2015). Potentially, this class of protein is not able to engage or be retained at PCH in cell types with lower levels of H3K9me3 such as ESCs. As PCH regulation and binding of pericentromeric-associated proteins are critical for centromere function in other cell types (Hall et al. 2012; Saksouk et al. 2015), it is possible that maintaining a particular PCH architecture may also be linked to preserving centromere function in ESCs. ESCs could have acquired a unique form of centromere organization along with other unusual properties of pluripotent cells, such as their cell cycle parameters, DNA damage checkpoints, or prolonged maintenance undergoing self-renewal (Burdon et al. 2002; Weissbein et al. 2014). Therefore, it will be important in future research to examine centromere function more closely in ESCs that have an experimentally perturbed heterochromatin organization. Last, future studies should also investigate how higher-order chromatin structure can influence nuclear organization and genome interactions in regions outside of heterochromatin in pluripotent cells; for instance, in coordinating movements of chromosome territories upon cell differentiation and reprogramming (Politz et al. 2013).

## Materials and methods

### Cell lines

E14Tg2a (129P2/OlaHsd; passages 19–28) (Hooper et al. 1987), J1 (129S4/SvJae; passages 20–24), EF1 (E14Tg2a-derived *Nanog*-overexpressing cells; passages 22–26) (Chambers et al. 2003), RCNβH (E14Tg2a-derived *Nanog*^+/–^; passages 40–44) (Chambers et al. 2007), RCNβH-B(t) (E14Tg2a-derived *Nanog*^–/–^; passages 20–30) (Chambers et al. 2007), TβC44cre6 (E14Tg2a-derived *Nanog*^–/–^; passages 32–36) (Chambers et al. 2007), TNGA (E14Tg2a-derived *Nanog*-*GFP* knock-in) (Chambers et al. 2007), and *Sall1*-del (E14.1-derived *Sall1*^–/–^; passages 20–30) (Nishinakamura et al. 2001; Yuri et al. 2009) ESCs were cultured on gelatin-coated surfaces in standard ESC medium (DMEM supplemented with 15% FBS, 1 mM sodium pyruvate, 0.1 mM 2-mercaptoethanol, 0.1 mM nonessential amino acids, 2 mM glutamax, 1000 U/mL LIF). All ESC lines were male. During expansion of RCNβH-B(t) ESCs, 25 µg/mL hygromycin was added to select for *Nanog*^–/–^ cells. Where indicated as 2i conditions, ESCs were cultured for more than five passages on gelatin-coated surfaces in N2B27 (1:1 DMEM/F-12:neurobasal, 2 mM glutamax, 0.1 mM 2-mercaptoethanol, 1% B27, 0.5% N2) supplemented with 1 µM PD0325901, 3 µM CHIR99021, and 1000 U/mL LIF. ESC differentiation was achieved by plating 300,000 cells onto a gelatin-coated 10-cm plate in ESC medium. After 24 h, medium was switched to ESC medium without LIF (supplemented with 5 µM all-*trans* retinoic acid) and changed daily.

Embryo-derived 129S2 (passages 14–28) (Brons et al. 2007) and B2 (ICR; passages 8–14) (Rugg-Gunn et al. 2012) EpiSCs were cultured on 10 µg/mL fibronectin or γ-irradiated mouse embryonic fibroblasts in N2B27 supplemented with 20 ng/mL Activin A and 12 ng/mL bFGF. Both EpiSC lines were female. *Sall1*^–/–^ EpiSCs were generated by converting *Sall1*^–/–^ ESCs into EpiSCs as described (Rugg-Gunn et al. 2012). *Sall1*^–/–^ EpiSCs were maintained for at least 10 passages in EpiSC conditions before use. See the Supplemental Material for a detailed description of the transgenic cell lines used.

### Imaging and analysis

Cells were cultured on glass coverslips precoated with gelatin, fibronectin, or γ-irradiated mouse embryonic fibroblasts. Samples for ESI were processed and analyzed as described (Ahmed et al. 2010). For the majority of immunofluorescent experiments, cells were fixed with 2% paraformaldehyde in PBS for 10 min at room temperature, washed three times with PBS for 5 min, and blocked with 5% FBS and 0.1% Triton X-100 in PBS for 1 h. Cells were incubated with primary antibody (Supplemental Material) in blocking buffer overnight at 4°C, washed three times with PBS for 5 min, and incubated with secondary antibodies for 2 h at room temperature. Nuclei were counterstained with DAPI. Images were collected on an Olympus FV1000 confocal microscope. Optical section thickness ranged from 0.5 to 2 µm. ImageJ software was used to quantify H3K9me3 foci size and intensity using the “analyze particles” tool. Line scan analysis was performed as described (Fussner et al. 2011).

## Supplementary Material

Supplemental Material
