# Supplementary material for: The pluripotency factor Nanog regulates pericentromeric heterochromatin organization in mouse embryonic stem cells
Source: Genes Dev. 2016 May 1;30(9):1101–15. doi: 10.1101/gad.275685.115 (PMC4863740; doi:10.1101/gad.275685.115)
Supplement: Supplemental Material [file supp_30_9_1101__index.html]

The pluripotency factor Nanog regulates pericentromeric heterochromatin organization in mouse embryonic stem cells — Supplemental Material 

# The pluripotency factor *Nanog* regulates pericentromeric heterochromatin organization in mouse embryonic stem cells

## Supplemental Material

**Files in this Data Supplement:**

- Supp Material.pdf
