# Supplementary material for: The pluripotency factor Nanog regulates pericentromeric heterochromatin organization in mouse embryonic stem cells
Source: Genes Dev. 2016 May 1;30(9):1101–15. doi: 10.1101/gad.275685.115 (PMC4863740; doi:10.1101/gad.275685.115)
Supplement: Supplemental Material [file supp_gad.275685.115_SuppMaterial.pdf]

**Supplemental Material**

**Novo et al.,**

**The pluripotency factor *Nanog* regulates pericentromeric heterochromatin organization in mouse embryonic stem cells**

List of material provided:

- Supplemental Fig. S1: related to Fig. 1. Additional evidence that *Nanog* is required for chromocentre organization in ESC.
- Supplemental Fig. S2: related to Fig. 1. Additional evidence that *Nanog*$^{-/-}$ ESC retain key indictors of undifferentiated state.
- Supplemental Fig. S3: related to Fig. 3. Additional evidence that *Nanog* is sufficient to induce chromocentre reorganization in EpiSC.
- Supplemental Fig. S4: related to Fig. 3. Additional evidence that alternative pluripotency factors are unable to remodel chromatin in EpiSC.
- Supplemental Fig. S5: related to Fig. 3. Additional evidence that changes in *Nanog* levels do not impact other repeat sequence classes or cell cycle parameters in ESC or EpiSC.
- Supplemental Fig. S6: related to Fig. 5. Additional characterization of *NanogΔC* and *TALE-CD2* EpiSC.
- Supplemental Fig. S7: related to Fig. 6. Additional evidence that *Sall1* is required for chromocentre organization in ESC.
- Additional details on Material and Methods.

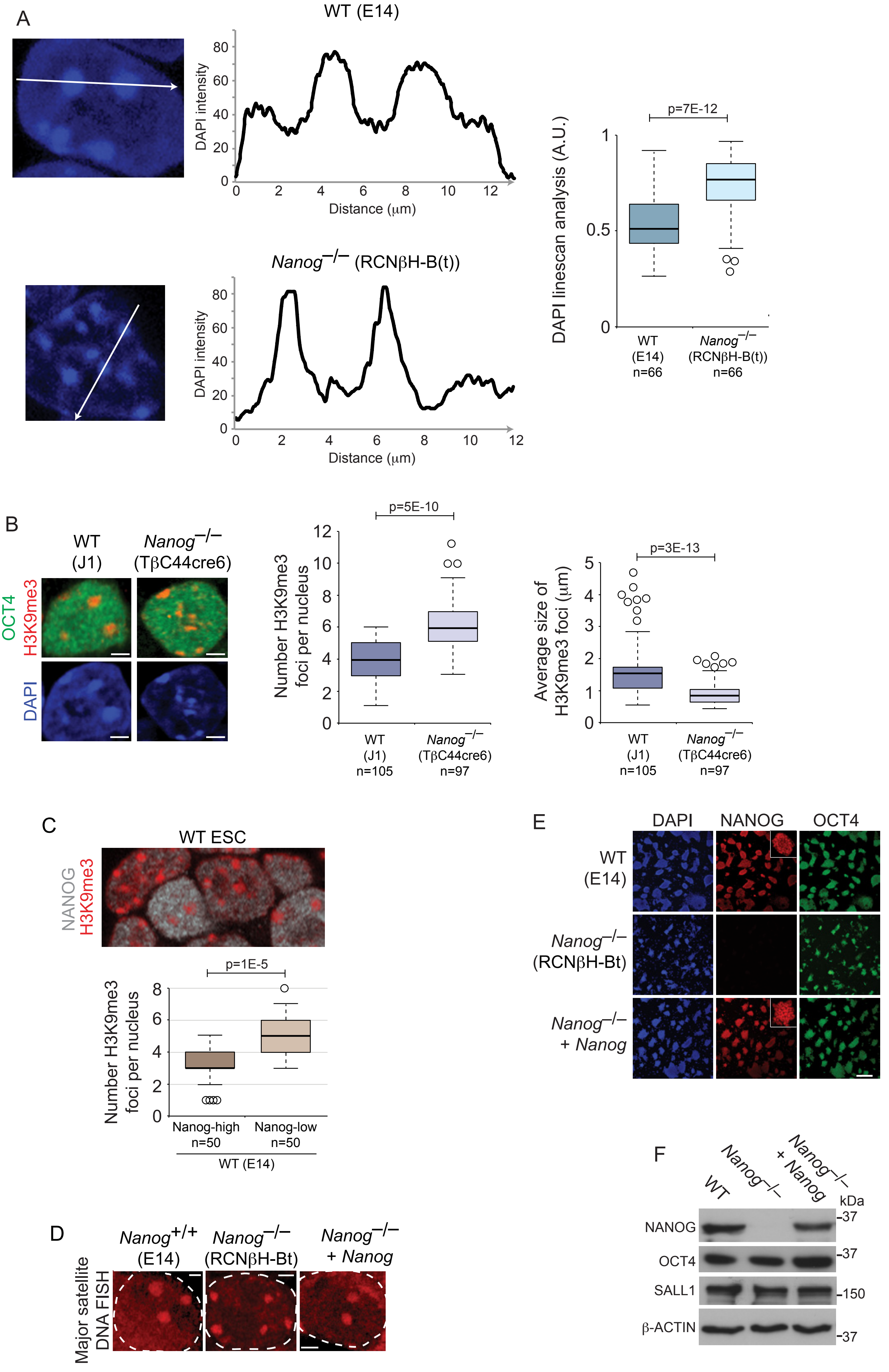
A
WT (E14)
DAPI intensity
Distance (µm)
Nanog−/− (RCNβH-B(t))
DAPI linescan analysis (A.U.)
p=7E-12
WT (E14) n=66
Nanog−/− (RCNβH-B(t)) n=66
B
WT (J1)
Nanog−/− (TβC44cre6)
OCT4 H3K9me3
DAPI
Number H3K9me3 foci per nucleus
p=5E-10
WT (J1) n=105
Nanog−/− (TβC44cre6) n=97
Average size of H3K9me3 foci (µm)
p=3E-13
C
WT ESC
NANOG H3K9me3
p=1E-5
Nanog-high n=50
Nanog-low n=50
WT (E14)
D
Nanog+/+ (E14)
Nanog−/− (RCNβH-Bt)
Nanog−/− + Nanog
Major satellite DNA FISH
E
DAPI
NANOG
OCT4
WT (E14)
Nanog−/− (RCNβH-Bt)
Nanog−/− + Nanog
F
WT
Nanog−/−
Nanog−/− + Nanog
kDa
NANOG
OCT4
SALL1
β-ACTIN
-37
-37
-150
-37

**Supplemental Figure S1, related to Fig. 1. *Nanog* is required for chromocentre organization in ESC.**

(A) DAPI linescan analysis of WT and *Nanog*$^{-/-}$ ESC. Box and whisker plots show the ratio of DAPI signal between nucleoplasmic background and chromocentre. Data were compared using a Student's t test.

(B) Chromocentre organization revealed by immunofluorescent analysis of H3K9me3, OCT4 and DAPI in alternative lines of WT (J1) and *Nanog*$^{-/-}$ ESC (TβC44cre6). Scale bar, 2μM. Box and whisker plots show the number (left) and size (right) of H3K9me3 foci per nucleus. Data were compared using a Student's t test.

(C) Chromocentre organization revealed by immunofluorescent analysis of H3K9me3 in high *Nanog*-expressing and low *Nanog*-expressing WT ESC.

(D) DNA FISH for major satellite repeats. Representative images from at least 50 nuclei collected for each cell line. A matched scrambled probe yielded no signal (data not shown). Scale bar, 2μm.

(E) Immunofluorescent images show NANOG and OCT4 localization in WT, *Nanog*$^{-/-}$ and *Nanog*$^{-/-}$ +*Nanog* ESC. Insets show expanded view of single colonies. Scale bar, 250μM.

(F) Western blot of WT, *Nanog*$^{-/-}$ and *Nanog*$^{-/-}$ + *Nanog* ESC with indicated antibodies.

Novo_FigS2

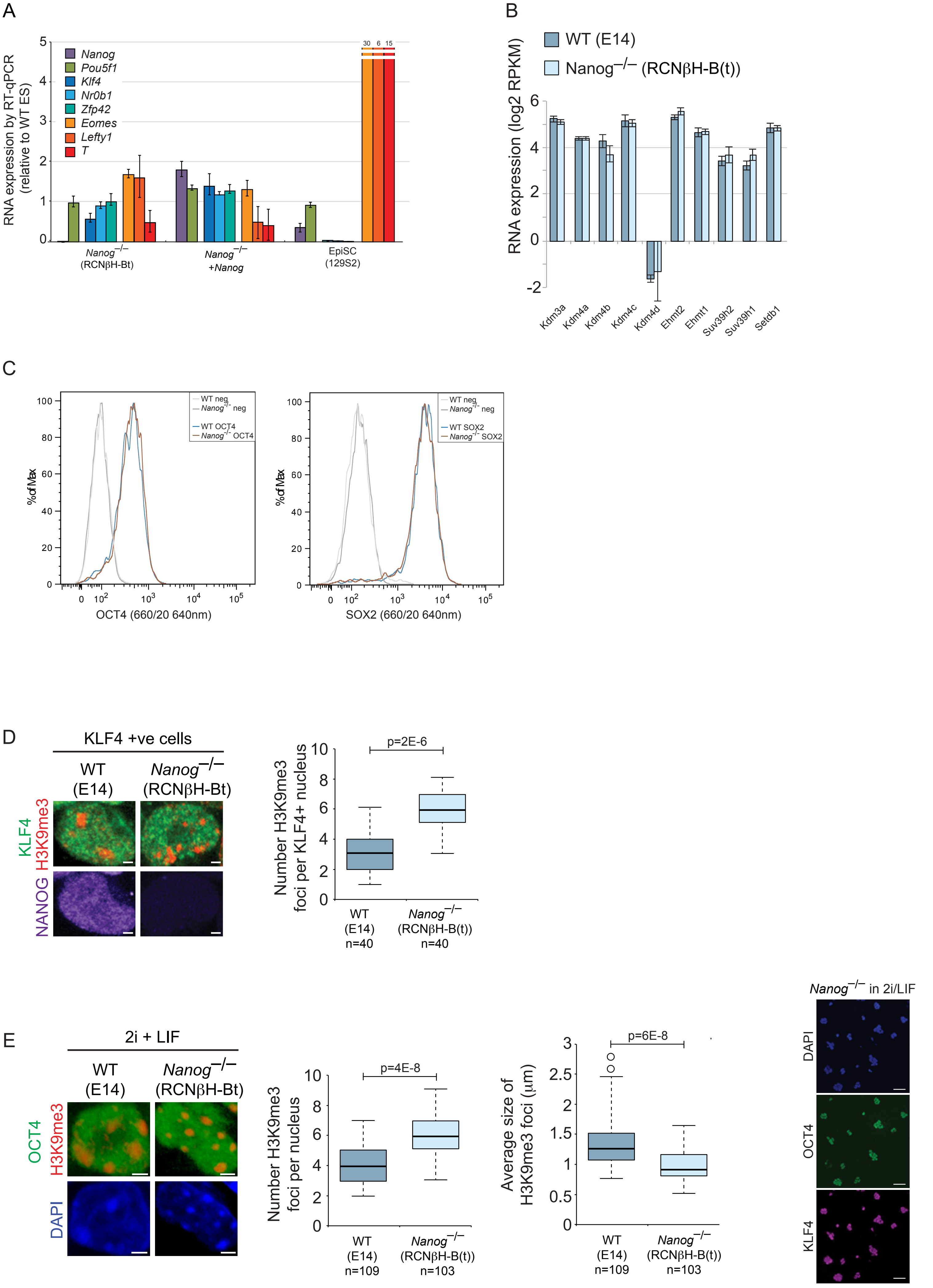
A
RNA expression by RT-qPCR (relative to WT ES)
Nanog
Pou5f1
Klf4
Nr0b1
Zfp42
Eomes
Lefty1
T
30 6 15
Nanog−/− (RCNβH-Bt)
Nanog−/− +Nanog
EpiSC (129S2)
B
WT (E14)
Nanog−/− (RCNβH-B(t))
RNA expression (log2 RPKM)
Kdm3a
Kdm4a
Kdm4b
Kdm4c
Kdm4d
Ehmt2
Ehmt1
Suv39h2
Suv39h1
Setdb1
C
WT neg
Nanog−/− neg
WT OCT4
Nanog−/− OCT4
% of Max
OCT4 (660/20 640nm)
WT neg
Nanog−/− neg
WT SOX2
Nanog−/− SOX2
SOX2 (660/20 640nm)
D
KLF4 +ve cells
WT (E14)
Nanog−/− (RCNβH-Bt)
KLF4 H3K9me3
NANOG
p=2E-6
Number H3K9me3 foci per KLF4+ nucleus
WT (E14) n=40
Nanog−/− (RCNβH-B(t)) n=40
E
2i + LIF
WT (E14)
Nanog−/− (RCNβH-Bt)
OCT4 H3K9me3
DAPI
p=4E-8
Number H3K9me3 foci per nucleus
WT (E14) n=109
Nanog−/− (RCNβH-B(t)) n=103
p=6E-8
Average size of H3K9me3 foci (µm)
WT (E14) n=109
Nanog−/− (RCNβH-B(t)) n=103
Nanog−/− in 2i/LIF
DAPI
OCT4
KLF4

**Supplemental Figure 2, related to Fig. 1. *Nanog$^{-/-}$* ESC retain key indictors of undifferentiated state.**

(A) RT-qPCR of pluripotency genes, *Nanog*, *Oct4*, *Klf4*, *Nr0b1*, *Zfp42*, and early differentiation genes, *Eomes*, *Lefty1* and *T*. Values were normalized to *Hmbs* and shown relative to WT ESC. EpiSC was included as a positive control for *Eomes*, *Lefty1* and *T*. Data represent mean ± SD from three biological replicates.

(B) RNA levels of H3K9me3 methyltransferases and histone demethylases are unchanged in the absence of *Nanog*. Data represent mean ± SD from three biological replicates.

(C) Intracellular flow cytometry reveals a similar distribution of OCT4 and SOX2 levels in WT and Nanog$^{-/-}$ ESC. Grey lines show isotype controls.

(D) Chromocentre organization revealed by immunofluorescent analysis of H3K9me3 in KLF4-positive WT and *Nanog$^{-/-}$* ESC. Scale bar, 2μM. Box and whisker plots show the number (left) and size (right) of H3K9me3 foci per nucleus. Data were compared using a Student's t test.

(E) Chromocentre organization revealed by immunofluorescent analysis of H3K9me3, OCT4 and DAPI in WT and *Nanog$^{-/-}$* ESC maintained in 2i+LIF conditions. Right panels show uniform expression of OCT4 and KLF4 in *Nanog$^{-/-}$* ESC maintained in 2i+LIF conditions. Scale bar, 50μm.

Novo_FigS3

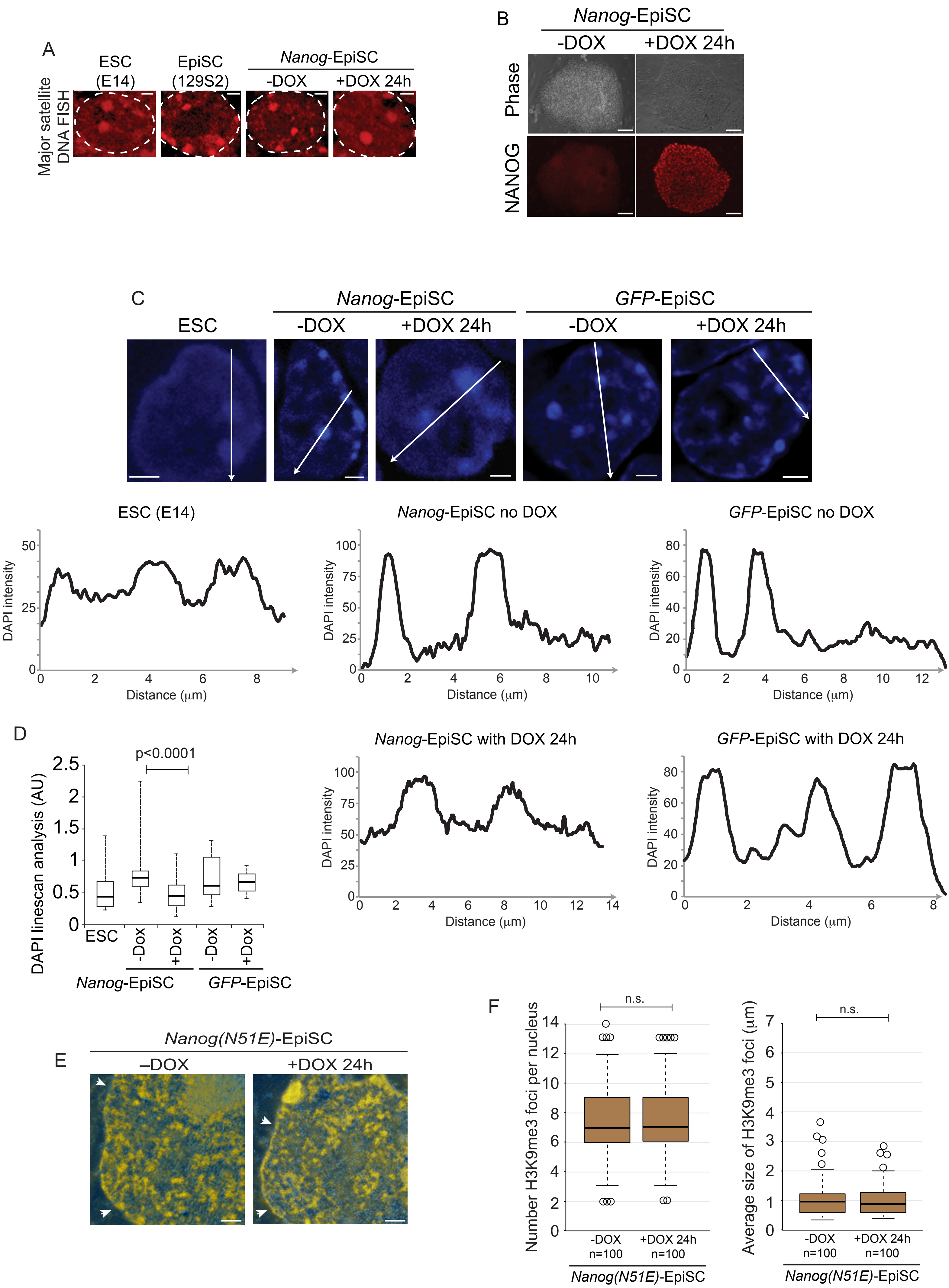
A
ESC (E14)
EpiSC (129S2)
Nanog-EpiSC
-DOX
+DOX 24h
Major satellite DNA FISH
B
Nanog-EpiSC
-DOX
+DOX 24h
Phase
NANOG
C
ESC
Nanog-EpiSC
-DOX
+DOX 24h
GFP-EpiSC
-DOX
+DOX 24h
ESC (E14)
Nanog-EpiSC no DOX
GFP-EpiSC no DOX
DAPI intensity
Distance (μm)
D
p<0.0001
DAPI linescan analysis (AU)
ESC
-Dox
+Dox
-Dox
+Dox
Nanog-EpiSC
GFP-EpiSC
Nanog-EpiSC with DOX 24h
GFP-EpiSC with DOX 24h
DAPI intensity
Distance (μm)
E
Nanog(N51E)-EpiSC
–DOX
+DOX 24h
F
n.s.
Number H3K9me3 foci per nucleus
-DOX n=100
+DOX 24h n=100
Nanog(N51E)-EpiSC
n.s.
Average size of H3K9me3 foci (μm)
-DOX n=100
+DOX 24h n=100
Nanog(N51E)-EpiSC

**Supplemental Figure 3, related to Fig. 3. *Nanog* is sufficient to induce chromocentre reorganization in EpiSC.**

(A) DNA FISH for major satellite repeats. DOX was applied for 24h. Representative images from at least 50 nuclei collected for each cell line.

(B) Brightfield image and immunofluorescent signal for NANOG shown in DOX-inducible *Nanog*-EpiSC. DOX was applied for 24h. Scale bar, 100μm.

(C) DAPI linescan analysis of WT ESC, *Nanog*-EpiSC and *GFP*-Nanog EpiSC with and without DOX-induction for 24h.

(D) Box and whisker plots show the ratio of DAPI signal between nucleoplasmic background and chromocentre.

(E) Representative ESI images reveal unchanged chromatin organization upon DOX-mediated induction of the DNA binding mutant *Nanog(N51E)* in EpiSC. DOX was applied for 24h. Nuclear membrane indicated with arrowhead. Scale bar, 0.5μm.

(F) Chromocentre organization revealed by immunofluorescent analysis of H3K9me3 in *Nanog(N51E)*-EpiSC with and without DOX-induction for 24h. Box and whisker plots show the number (left) and size (right) of H3K9me3 foci per nucleus. Data were compared using a Student's t test.

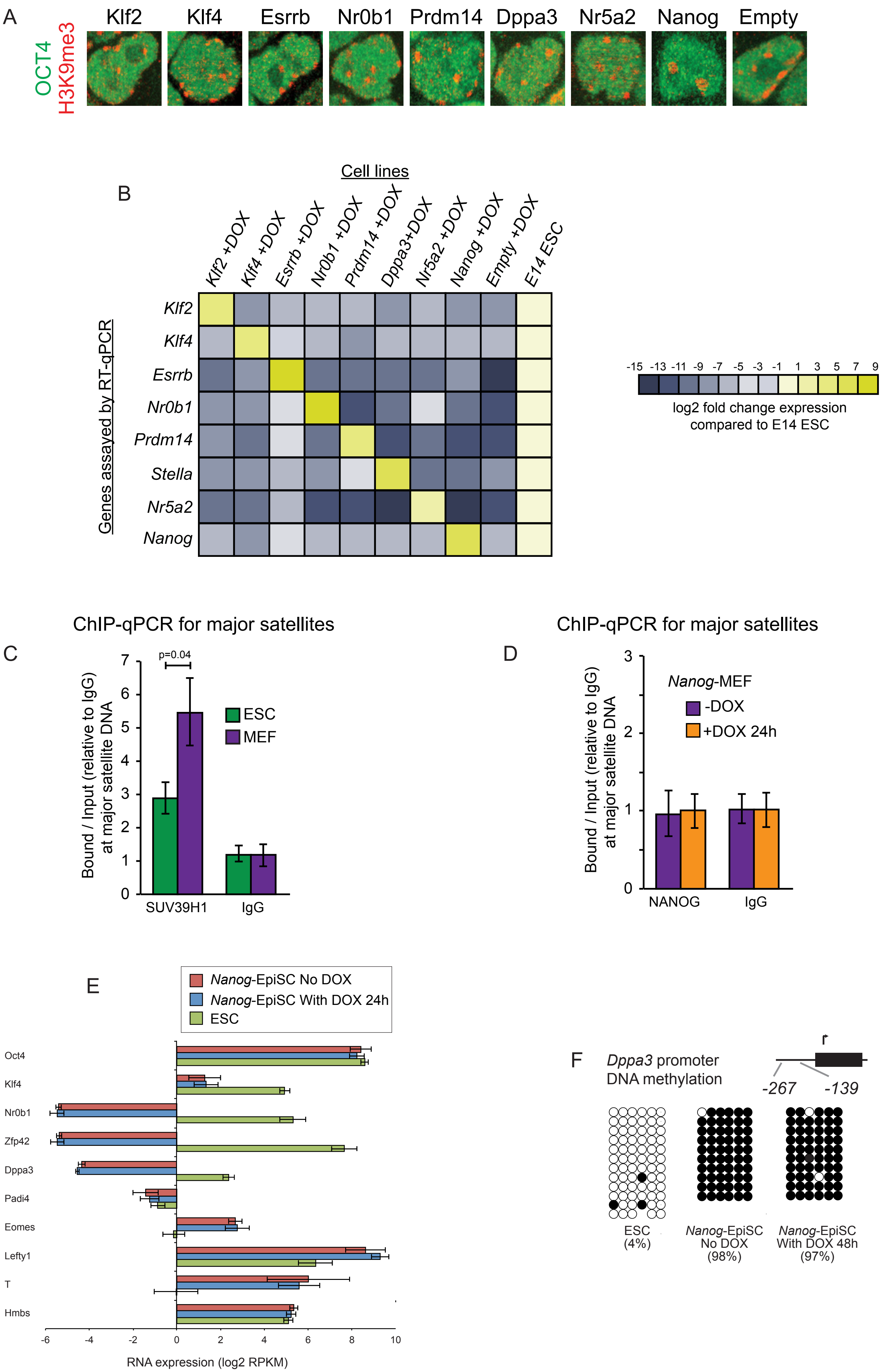
A
Klf2
Klf4
Esrrb
Nr0b1
Prdm14
Dppa3
Nr5a2
Nanog
Empty
OCT4
H3K9me3
B
Cell lines
Klf2 +DOX
Klf4 +DOX
Esrrb +DOX
Nr0b1 +DOX
Prdm14 +DOX
Dppa3+DOX
Nr5a2 +DOX
Nanog +DOX
Empty +DOX
E14 ESC
Genes assayed by RT-qPCR
Klf2
Klf4
Esrrb
Nr0b1
Prdm14
Stella
Nr5a2
Nanog
-15 -13 -11 -9 -7 -5 -3 -1 1 3 5 7 9
log2 fold change expression compared to E14 ESC
C
ChIP-qPCR for major satellites
Bound / Input (relative to IgG) at major satellite DNA
p=0.04
ESC
MEF
SUV39H1
IgG
D
ChIP-qPCR for major satellites
Nanog-MEF
-DOX
+DOX 24h
NANOG
IgG
E
Nanog-EpiSC No DOX
Nanog-EpiSC With DOX 24h
ESC
Oct4
Klf4
Nr0b1
Zfp42
Dppa3
Padi4
Eomes
Lefty1
T
Hmbs
RNA expression (log2 RPKM)
F
Dppa3 promoter DNA methylation
-267
-139
ESC (4%)
Nanog-EpiSC No DOX (98%)
Nanog-EpiSC With DOX 48h (97%)

**Supplemental Figure 4, related to Fig. 3. Alternative pluripotency factors are unable to remodel chromatin in EpiSC.**

(A) Chromocentre organization revealed by immunofluorescent analysis of H3K9me3 in EpiSC that overexpressed indicated gene for 24h using a DOX-inducible plasmid. Out of the factors tested, only *Nanog* was able to remodel chromocentre organization. Quantitation shown in Fig. 3D.

(B) RT-qPCR analysis of the various EpiSC lines after 24h DOX-induction, relative to WT ESC.

(C) ChIP-qPCR for SUV39H1 and IgG at major satellite DNA in WT ESC and MEF. Values were normalized to IgG. Data represent mean ± SD from three biological experiments.

(D) ChIP-qPCR for NANOG and IgG at major satellite DNA in *Nanog*-MEF with and without 24h DOX-induction. Values were normalized to IgG. Data represent mean ± SD from three biological experiments.

(E) Gene expression analysis reveals unchanged transcript levels after 24h DOX-induction in *Nanog*-EpiSC. Genes analyzed include those expressed highly in ESC relative to EpiSC (*Klf4*; *Nr0b1*; *Zfp42*), highly in EpiSC relative to ESC (*Eomes*; *Lefty1*; *T*) and controls (*Oct4*; *Hmbs*). Data represent mean ± SD for three biological replicates.

(F) DNA methylation analysis of the *Dppa3* promoter, which differs between ESC and EpiSC, revealed no change after *Nanog* induction, with the promoter remaining highly methylated. Open circles, unmethylated; filled circles, methylated. Position in base pairs from transcriptional start site indicated.

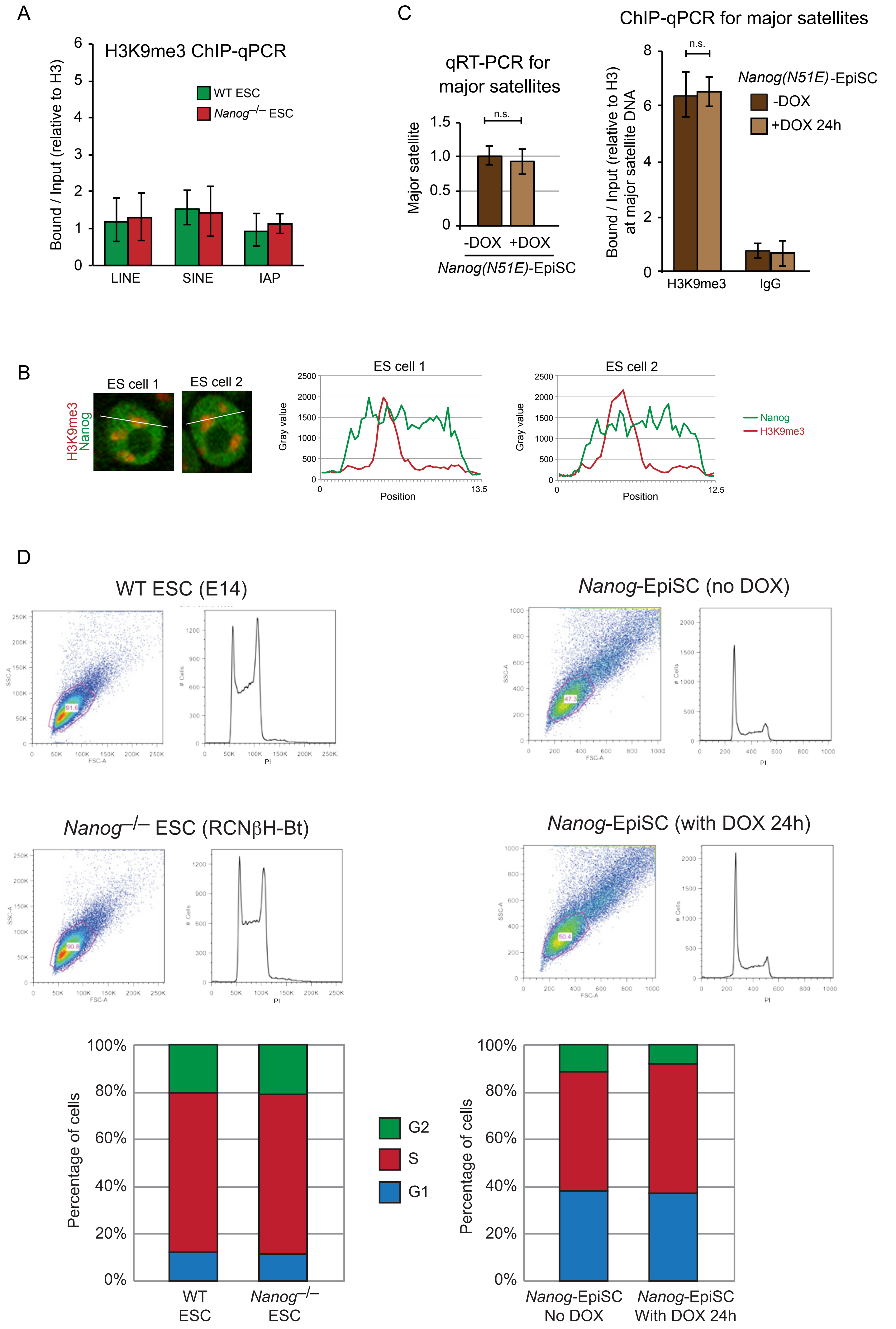
A
H3K9me3 ChIP-qPCR
WT ESC
Nanog−/− ESC
Bound / Input (relative to H3)
LINE
SINE
IAP
C
qRT-PCR for major satellites
Major satellite
n.s.
-DOX +DOX
Nanog(N51E)-EpiSC
ChIP-qPCR for major satellites
Bound / Input (relative to H3) at major satellite DNA
Nanog(N51E)-EpiSC
-DOX
+DOX 24h
H3K9me3
IgG
B
ES cell 1
ES cell 2
H3K9me3
Nanog
Gray value
Position
Nanog
H3K9me3
D
WT ESC (E14)
Nanog-EpiSC (no DOX)
Nanog−/− ESC (RCNβH-Bt)
Nanog-EpiSC (with DOX 24h)
SSC-A
FSC-A
# Cells
PI
Percentage of cells
G2
S
G1
WT ESC
Nanog−/− ESC
Nanog-EpiSC No DOX
Nanog-EpiSC With DOX 24h

**Supplemental Figure 5, related to Fig. 3. Changes in *Nanog* levels do not impact other repeat sequence classes or cell cycle parameters in ESC or EpiSC.**

(A) ChIP-qPCR for H3K9me3 LINE, SINE and IAP DNA in WT ESC and *Nanog*$^{-/-}$ ESC. Values were normalized to unmodified H3. Data represent mean ± SD from three biological experiments.

(B) Immunofluorescent microscopy and linescan analysis of NANOG and H3K9me3 in WT ESC.

(C) RT-qPCR for major satellite transcripts in *Nanog(N51E)*-EpiSC with and without DOX-induction for 24h (left). Values were normalized to *Hmbs*. ChIP-qPCR for H3K9me3 and IgG (normalized to unmodified H3) at major satellite DNA in *Nanog(N51E)*-EpiSC with and without DOX-induction for 24h (right). Data represent mean ± SD from three biological experiments.

(D) Flow cytometry scatter plots (forward vs side scatter) and propidium iodide (PI) histograms in WT ESC, *Nanog*$^{-/-}$ ESC, *Nanog*-EpiSC with and without 24h DOX-induction. Shown underneath are stacked charts revealing the percentage of cells in G1 / S /G2 for each cell type.

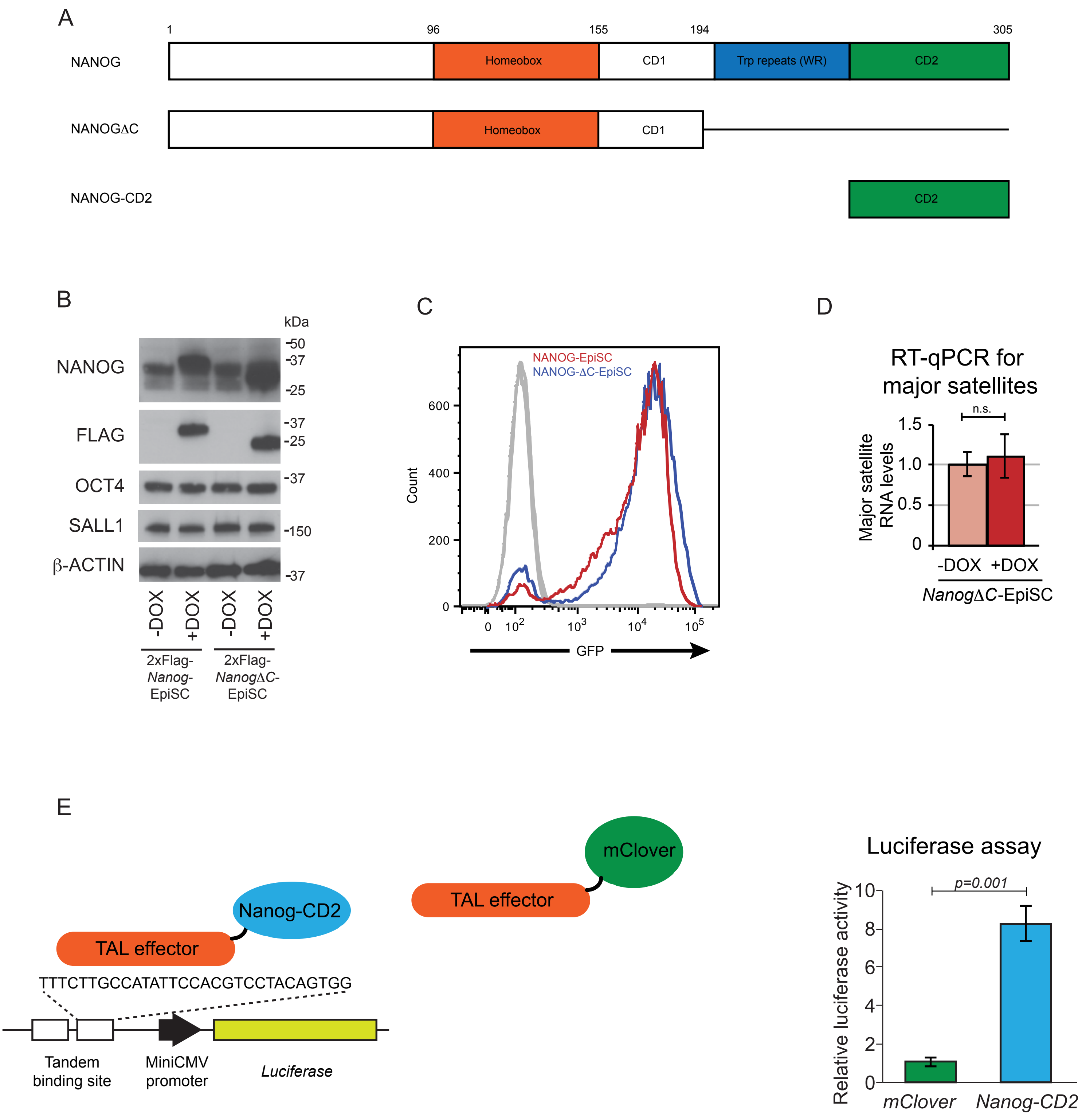
A
1
96
155
194
305
NANOG
Homeobox
CD1
Trp repeats (WR)
CD2
NANOGΔC
Homeobox
CD1
NANOG-CD2
CD2
B
kDa
NANOG
FLAG
OCT4
SALL1
β-ACTIN
-50
-37
-25
-37
-25
-37
-150
-37
-DOX
+DOX
-DOX
+DOX
2xFlag-Nanog-EpiSC
2xFlag-NanogΔC-EpiSC
C
NANOG-EpiSC
NANOG-ΔC-EpiSC
Count
600
400
200
0
0
10^2
10^3
10^4
10^5
GFP
D
RT-qPCR for major satellites
n.s.
Major satellite RNA levels
1.5
1.0
0.5
0
-DOX
+DOX
NanogΔC-EpiSC
E
Nanog-CD2
TAL effector
TTTCTTGCCATATTCCACGTCCTACAGTGG
Tandem binding site
MiniCMV promoter
Luciferase
mClover
TAL effector
Luciferase assay
p=0.001
Relative luciferase activity
10
8
6
4
2
0
mClover
Nanog-CD2

**Supplemental Figure 6, related to Fig. 5. Characterization of NanogΔC and TALE-CD2 EpiSC.**

(A) NANOG domain structure.

(B) Western blot of 2xFLAG-*Nanog*-EpiSC and 2xFLAG-*NanogΔC*-EpiSC with and without 24h DOX-induction.

(C) 2xFLAG-*Nanog* and 2xFLAG-*NanogΔC* transgenes were linked via IRES to GFP cDNA as a means to monitor induction levels in EpiSC. Flow cytometry analysis of GFP expression reveals similar levels of induction for the 2xFLAG-*Nanog* and 2xFLAG-*NanogΔC* plasmids after 24h DOX-induction. Grey lines indicate no DOX controls.

(D) RT-qPCR for major satellite transcripts in *NanogΔC*-EpiSC. Values were normalized to *Hmbs* and shown relative to WT ESC.

(E) Diagram of TALE-CD2 and TALE-mClover fusion proteins and luciferase reporter plasmid (left) and increase in luciferase activity upon expression of TALE-Nanog-CD2 compared to TALE-mClover.

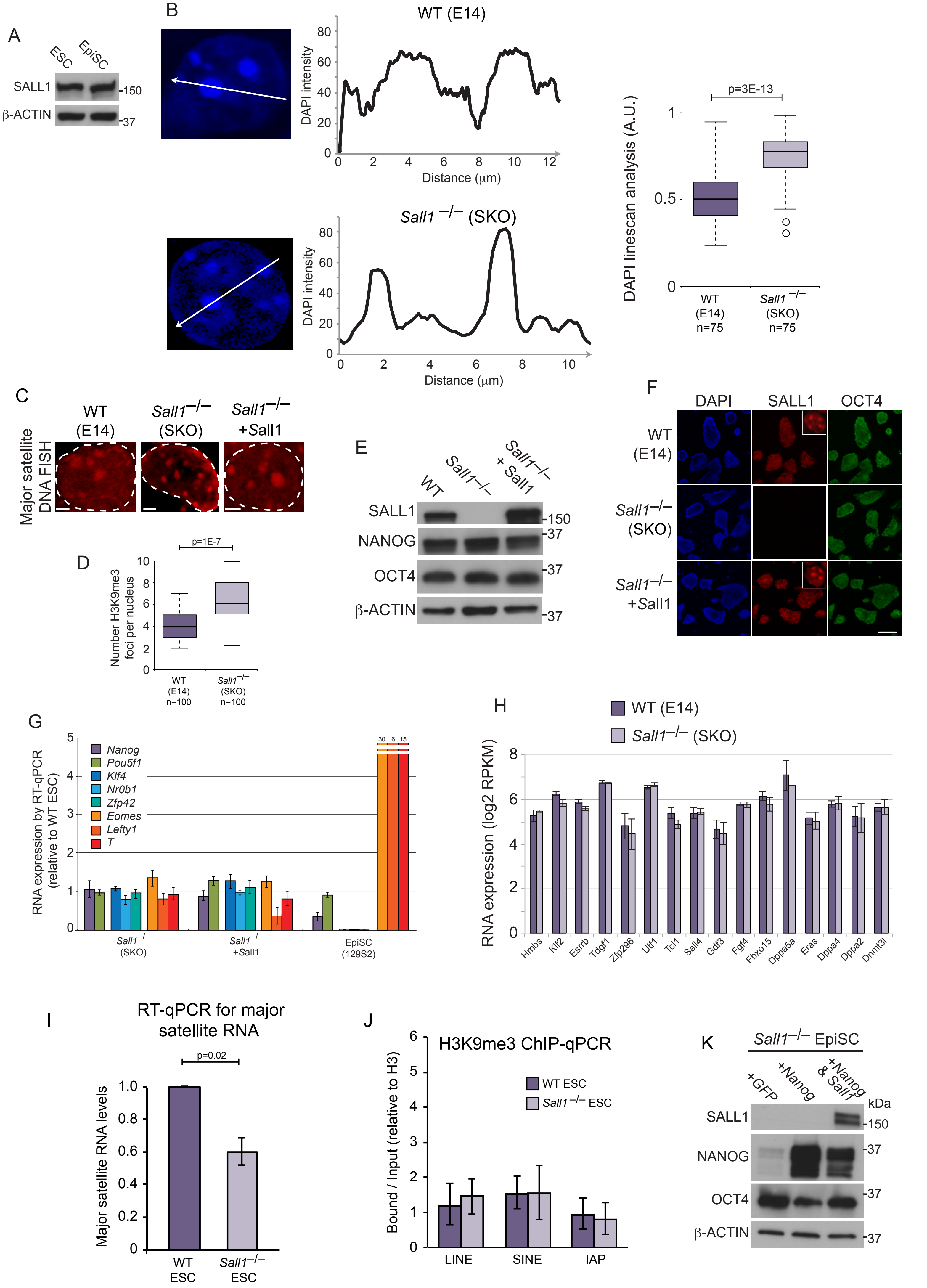
A
ESC
EpiSC
SALL1
β-ACTIN
150
37
B
WT (E14)
DAPI intensity
Distance (μm)
Sall1 −/− (SKO)
DAPI linescan analysis (A.U.)
p=3E-13
WT (E14) n=75
Sall1 −/− (SKO) n=75
C
WT (E14)
Sall1−/− (SKO)
Sall1−/− +Sall1
Major satellite DNA FISH
D
p=1E-7
Number H3K9me3 foci per nucleus
WT (E14) n=100
Sall1−/− (SKO) n=100
E
WT
Sall1−/−
Sall1−/− + Sall1
SALL1
NANOG
OCT4
β-ACTIN
150
37
F
DAPI
SALL1
OCT4
WT (E14)
Sall1−/− (SKO)
Sall1−/− +Sall1
G
RNA expression by RT-qPCR (relative to WT ESC)
Nanog
Pou5f1
Klf4
Nr0b1
Zfp42
Eomes
Lefty1
T
30
6
15
Sall1−/− (SKO)
Sall1−/− +Sall1
EpiSC (129S2)
H
WT (E14)
Sall1−/− (SKO)
RNA expression (log2 RPKM)
Hmbs
Klf2
Esrrb
Tdgf1
Zfp296
Utf1
Tcl1
Sall4
Gdf3
Fgf4
Fbxo15
Dppa5a
Eras
Dppa4
Dppa2
Dnmt3l
I
RT-qPCR for major satellite RNA
p=0.02
Major satellite RNA levels
WT ESC
Sall1−/− ESC
J
H3K9me3 ChIP-qPCR
Bound / Input (relative to H3)
WT ESC
Sall1−/− ESC
LINE
SINE
IAP
K
Sall1−/− EpiSC
+GFP
+Nanog
+Nanog & Sall1
kDa
SALL1
NANOG
OCT4
β-ACTIN
150
37

**Supplemental Figure 7, related to Fig. 6. *Sall1* is required for chromocentre organization in ESC.**

(A) Western blot shows similar levels of SALL1 in ESC and EpiSC.

(B) DAPI linescan analysis of WT and *Sall1*$^{-/-}$ ESC. Box and whisker plots show the ratio of DAPI signal between nucleoplasmic background and chromocentre. Data were compared using a Student's t test.

(C) DNA FISH for major satellite repeats. Representative images from at least 50 nuclei collected for each cell line. Scale bar, 2μm.

(D) Chromocentre organization revealed by immunofluorescent analysis of H3K9me3 in WT and *Sall1*$^{-/-}$ ESC maintained in 2i+LIF conditions.

(E) Western blot of WT, *Sall1*$^{-/-}$and *Sall1*$^{-/-}$ + *Sall1* ESC with indicated antibodies.

(F) Immunofluorescent images show SALL1 and OCT4 localization in WT, *Sall1*$^{-/-}$and *Sall1*$^{-/-}$ + *Sall1* ESC. Insets show expanded view of an individual cell, revealing heterochromatin localization of SALL1 signal. Scale bar, 250μM.

(G) RT-qPCR of pluripotency genes, *Nanog*, *Oct4*, *Klf4*, *Nr0b1*, *Zfp42*, and early differentiation genes, *Eomes*, *Lefty1* and *T*. Values were normalized to *Hmbs* and shown relative to WT ESC. EpiSC was included as a positive control for *Eomes*, *Lefty1* and *T*. Data represent mean ± SD from three biological replicates.

(H) RNA levels of ECATs are unchanged in the absence of *Sall1*. Data represent mean ± SD from three biological replicates.

(I) RT-qPCR for major satellite transcripts in WT and *Sall1*$^{-/-}$ ESC. Values were normalized to *Hmbs* and shown relative to WT ESC.

(J) ChIP-qPCR for H3K9me3 LINE, SINE and IAP DNA in WT ESC and *Sall1*$^{-/-}$ ESC. Values were normalized to unmodified H3. Data represent mean ± SD from three biological experiments.

(K) Western blot analysis of *Sall1*$^{-/-}$ EpiSC after 24h induction of *GFP*, *Nanog*, or *Nanog* and *Sall1*.

## Additional details on Material and Methods

### Cell transfections

All transfections were achieved using Lipofectamine 2000. *Nanog*$^{-/-}$ + *Nanog* and *Sall1*$^{-/-}$ + *Sall1* ESC were generated by transfecting 4μg pCAG-*Nanog*-IRES-*Puro* or 4μg pCAG-*Sall1*-IRES-*Puro* plasmids into *Nanog*$^{-/-}$ ESC or *Sall1*$^{-/-}$ ESC, followed by 1μg/ml puromycin selection and clonal expansion. *Nanog*-EpiSC were generated by transfecting 129S2 EpiSC with 1μg PB-TET-*Nanog*-IRES-*GFP*, 1μg pCAG-*rtTA-Puro* and 2μg pCyL43 piggyBac transposase (Wang et al. 2008), followed by selection with 1.2μg/ml puromycin. Individual clones were expanded and screened for efficient *Nanog* expression upon DOX-induction. Three clonal lines were used for this study. *Nanog(N51E)*-EpiSC were generated in the same way using *Nanog* cDNA with an N to E substitution at position 51 (Jauch et al., 2008). *NanogΔC*-EpiSC were generated in the same way using *Nanog* cDNA lacking amino acids 196 to 305 encoding the WR and CD2 domains. *GFP*-EpiSC, 2xFLAG-*Nanog*-EpiSC and 2xFLAG-*NanogΔC*-EpiSC were generated using PB-TET-GFP-ires-GFP, PB-TET-2xFLAG-*Nanog*-ires-GFP and PB-TET-2xFLAG-*NanogΔC*-ires-GFP plasmids, respectively. *Sall1*$^{-/-}$ EpiSC were transfected with PB-TET-*Nanog*-IRES-*GFP* and PB-TET-*Nanog*-IRES-*Sall1* plasmids and processed as described above. *Nanog*$^{-/-}$ ESC were transfected with PB-TET-2xFLAG-*Nanog*-ires-GFP and processed as described above. TALE-CD2 was generated by cloning the *Nanog* CD2 domain (amino acids 249 to 305) in frame into pTALYM3B15, replacing the mClover sequence (Miyanari et al. 2013). TALE-CD2 was cloned into PB-TET-ires-GFP; TALE-mClover was cloned into PB-TET-ires-Cherry. Sequences cloned into the PB-TET transposon plasmid are under the transcriptional control of the tet02 DOX-inducible promoter (Agha-Mohammadi et al. 2004). DOX was applied at 1μg/ml for 24h unless specified otherwise.

### DNA FISH

DNA FISH was performed essentially as described (Meshorer et al. 2006). Cells were fixed with 3.7% paraformaldehyde for 15min at room temperature, washed three times with PBS for 5min and permeabilized on ice for 5min with 0.5% Triton X-100 in PBS. Cells were washed three times with PBS for 5min and twice with 2xSSC for 5min. Probes (500ng) were denatured for 8min at 90°C in 50% formamide, 10% dextran sulphate,

1mg/ml yeast tRNA in 2xSSC, then kept on ice. Cells were denatured in 70% formamide / 2xSSC for 7min at 85°C, washed twice with cold 2xSSC and incubated overnight at 37°C with denatured probe. Cells were washed four times with 2xSSC for 20min, four times with 4xSSC for 10min, blocked with 3% BSA in 4xSSC for 30min and washed once with 4xSSC for 10min. Secondary antibody (Streptavidin, Alexa Fluor 555; Life Technologies) was applied for 1h in 4xSSC, then cells were washed four times with 4xSSC for 15min. Images were collected on an Olympus FV1000 confocal microscope. Optical section thickness ranged from 0.5-2μm. Probe sequences were Major Satellite [biotin]CTCGCCATATTTCACGTCCTAAAGTGTGTATTTCTC and Scrambled [biotin]TCTACGTTACCATCTCAGTGCGTATCGTTCTATTCA.

**Western blotting**

Whole cell lysates were prepared in RIPA buffer following standard protocols. Histone proteins were acid-extracted as described (Tavares et al. 2012). SDS-PAGE, transfer and detection were performed as previously described (Rugg-Gunn et al. 2010).

**RT-qPCR**

For most RT-qPCR experiments, RNA extracted with the RNeasy Micro Kit (Qiagen), reverse transcribed using the QuantiTect RT Kit (Qiagen) and subjected to quantitative PCR analysis as previously described (Rugg-Gunn et al. 2010). For analysis of major satellite and other repeat classes, total RNA was extracted with TRIzol (Life Technologies) and treated with two rounds of 1U DNase (Fermentas) per 1μg RNA to remove genomic DNA in the presence of RiboLock RNase inhibitor (Fermentas). RNA (1μg) was reverse transcribed using Superscript II (Life Technologies) and random primers (Promega). cDNA was amplified with SYBR Green Jump Start Taq Ready Mix (Sigma) using primers from Lehnertz et al. (2003) and Bulut-Karslioglu et al. (2012).

**Antibodies**

| Antibody | Company | Catalogue | IF dilution | WB dilution |
|---|---|---|---|---|
| β-actin | Sigma | A5441 | | 1 in 5000 |
| FLAG | Sigma | F3165 | | 1 in 2000 |

| | | | | |
|---|---|---|---|---|
| H3 | Abcam | ab1791 | | |
| H3K9Ac | Millipore | 07-352 | | |
| H3K9me3 | Abcam | ab8898 | | |
| H3K9me3 | Prim Singh (Cowell et al. 2002) | | 1 in 500 | |
| Klf4 | Santa Cruz | sc-20691 | 1 in 400 | |
| Nanog | Reprocell | RCAB0002 | 1 in 200 | 1 in 1000 |
| Nanog | eBioscience | 14-576-80 | | 1 in 500 |
| Oct4 | Santa Cruz | sc5279 | 1 in 100 | 1 in 500 |
| Oct4 | Abcam | ab19857 | 1 in 100 | |
| Sall1 | R&D | PP-K9814 | 1 in 200 | 1 in 1000 |
| Sall1 | Abcam | ab31526 | 1 in 400 | 1 in 500 |
| Sox2 | R&D | MAB2018 | 1 in 200 | |
| Suv39h1 | Abcam | Ab12405 | | |

**Immunoprecipitation and mass spectrometry**

Immunoprecipitations from nuclear extracts (Dignam et al. 1983) or a mixture of recombinant proteins (see below) using 2μg of NANOG antibody (Reprocell) or 2μg rabbit IgG (Jackson ImmunoResearch) crosslinked to 40μl Protein A magnetic beads (Life Technologies) as described (Tavares et al. 2012). Immunoprecipitations were performed either without treatment or in the presence of 25U benzonase and/or 2.5μg/ml ethidium bromide for three hours. Proteins were eluted from beads by incubation with SDS-loading dye for 1 hour at room temperature and analyzed by SDS-PAGE. Purification of 2xFLAG-NANOG from nuclear extract was carried out as previously described (van den Berg et al. 2010). Eluted proteins were analyzed by mass spectrometry as described (Iurlaro et al. 2013). Criteria for protein identification were: minimum of two peptides, each with a probability of >50% and an overall protein probability of >99%, which gave a protein false discovery rate of 0.1%.

**Recombinant NANOG and SALL1**

Full-length his-tagged NANOG and SALL1 were cloned into pDEST8 using the Gateway cloning system (Life Technologies). Recombinant Baculovirus were generated using the Bac-to-Bac system (Life Technologies) and used to infect Sf9 cells for 60h. NANOG and

SALL1 were purified as described previously (Elderkin et al. 2007). His-tagged NANOG wild-type and N51A homeodomain proteins were expressed in BL21(DE3). Cells were induced with 1mM IPTG at 30°C for 3hours. Protein was purified on Ni-NTA resin (Sigma) and dialyzed into 25mM Tris pH8.0, 500mM NaCl.

**Electrophoretic mobility shift assays**

Oligonucleotides were labelled at the 5' end of the upper strand with IR700 dye. Protein was added to oligonucleotide and buffer to give final concentrations of 10mM Hepes pH7.9, 10% glycerol, 50mM KCl, 10mM NaCl, 0.4mM EDTA, 2.5mM DTT, 0.25% Tween 20, 100μg/ml dI:dc, 1mg/ml BSA, 10nM oligonucleotide. Reactions were incubated at room temperature for 30 minutes before analysis on 5% acrylamide gels. Data were collected on a Licor Odyssey Fc. DNA probes were used corresponding to full-length major satellite repeat (234bp; Bulut-Karslioglu et al., 2012) and the NANOG-binding site of the *Tcf3* promoter: CTGTTAATGGGAGC (Jauch et al., 2008). The repeat probe was generated using the following primers: 5' GGACCTGGAATATGGCGAGAAAACTGAAAATAACG and 3' TTCAGTGGGCATTTCTCATTTTTC.

**DNA methylation analysis of *Dppa3* promoter**

Was performed as described (Rugg-Gunn et al. 2012).

**Cell cycle analysis**

One million cells were suspended in 200μl PBS, fixed by the drop wise addition of 2ml ice-cold 70% ethanol / 30% water, and incubated at 4°C for 1 hour. Cells were collected by centrifugation at 300g for 5min and resuspended in 500μl PBS. RNase A (100μl of 1mg/ml) was added and incubated for 10min at room temperature. Propidium iodide (50μl of 0.5mg/ml in 0.6% NP40 / PBS) was added and incubated for one hour at 37°C. Cells were analyzed on an LSRII Flow Cytometer with 50,000 events collected. Data were analyzed using FlowJo software (TreeStar) using the Dean-Jett-Fox Cell Cycle analysis model.

**Chromatin immunoprecipitation**

ChIP experiments for NANOG and SALL1 were performed as described (Tavares et al. 2012). Briefly, cells were fixed in 2mM DSG (Sigma) for 45min and then in 1% formaldehyde for 12min. Sonicated chromatin (250µg; 200-500bp fragments) were pre-cleared with blocked beads for 2h at 4°C and incubated at 4°C overnight with either 5µg Nanog antibody (Reprocell), 5µg Sall1 antibody (Abcam), 5µg Suv39h1 antibody (Abcam), or 5µg rabbit IgG (Jackson ImmunoResearch). Chromatin-antibody complexes were incubated for 6-8 hours at 4°C with protein A or G magnetic Dynabeads (Life Technologies), washed and crosslinks reversed. For Re-ChIP experiments, the following modifications to the above protocol were applied: 25µg of antibody was added to 20 million cells and the first ChIP was eluted with 20mM DTT 1% SDS for 30min at 37°C. Before the elute was diluted 50-fold in buffer, then 25µg of the second antibody was added and the above ChIP protocol was resumed. ChIP experiments for H3K9me3, H3K9ac and H3 were performed as described (Rugg-Gunn et al. 2010). ChIP DNA was analysed by qPCR using primers from Lehnertz et al. (2003) and Bulut-Karslioglu et al. (2012).

**Supplemental References**

Agha-Mohammadi S, O'Malley M, Etemad A, Wang Z, Xiao X, Lotze MT. 2004. Second-generation tetracycline-regulatable promoter: repositioned tet operator elements optimize transactivator synergy while shorter minimal promoter offers tight basal leakiness. *J Gene Med* **6**: 817-828.

Bulut-Karslioglu A, Perrera V, Scaranaro M, de la Rosa-Velazquez IA, van de Nobelen S, Shukeir N, Popow J, Gerle B, Opravil S, Pagani M et al. 2012. A transcription factor-based mechanism for mouse heterochromatin formation. *Nat Struct Mol Biol* **19**: 1023-1030.

Cowell IG, Aucott R, Mahadevaiah SK, Burgoyne PS, Huskisson N, Bongiorni S, Prantera G, Fanti L, Pimpinelli S, Wu R et al. 2002. Heterochromatin, HP1 and methylation at lysine 9 of histone H3 in animals. *Chromosoma* **111**: 22-36.

Dignam JD, Lebovitz RM, Roeder RG. 1983. Accurate transcription initiation by RNA polymerase II in a soluble extract from isolated mammalian nuclei. *Nuc Acids Res* **11**: 1475-1489.

Elderkin S, Maertens GN, Endoh M, Mallery DL, Morrice N, Koseki H, Peters G, Brockdorff N, Hiom K. 2007. A phosphorylated form of Mel-18 targets the Ring1B histone H2A ubiquitin ligase to chromatin. *Mol Cell* **28**: 107-120.

Iurlaro M, Ficz G, Oxley D, Raiber EA, Bachman M, Booth MJ, Andrews S, Balasubramanian S, Reik W. 2013. A screen for hydroxymethylcytosine and formylcytosine binding proteins suggests functions in transcription and chromatin regulation. *Genome Biol* **14**: R119.

Jauch R, Ng CK, Saikatendu KS, Stevens RC, Kolatkar PR. 2008. Crystal structure and DNA binding of the homeodomain of the stem cell transcription factor Nanog. J Mol Biol **376**:758-70.

Lehnertz B, Ueda Y, Derijck AA, Braunschweig U, Perez-Burgos L, Kubicek S, Chen T, Li E, Jenuwein T, Peters AH. 2003. Suv39h-mediated histone H3 lysine 9 methylation directs DNA methylation to major satellite repeats at pericentric heterochromatin. *Curr Biol* **13**: 1192-1200.

Meshorer E, Yellajoshula D, George E, Scambler PJ, Brown DT, Misteli T. 2006. Hyperdynamic plasticity of chromatin proteins in pluripotent embryonic stem cells. *Dev Cell* **10**: 105-116.

Miyanari Y, Ziegler-Birling C, Torres-Padilla ME. 2013. Live visualization of chromatin dynamics with fluorescent TALEs. *Nat Struct Mol Biol* **20**: 1321-1324.

Rugg-Gunn PJ, Cox BJ, Lanner F, Sharma P, Ignatchenko V, McDonald AC, Garner J, Gramolini AO, Rossant J, Kislinger T. 2012. Cell-surface proteomics identifies lineage-specific markers of embryo-derived stem cells. *Dev Cell* **22**: 887-901.

Rugg-Gunn PJ, Cox BJ, Ralston A, Rossant J. 2010. Distinct histone modifications in stem cell lines and tissue lineages from the early mouse embryo. *PNAS USA* **107**: 10783-10790.

Tavares L, Dimitrova E, Oxley D, Webster J, Poot R, Demmers J, Bezstarosti K, Taylor S, Ura H, Koide H et al. 2012. RYBP-PRC1 complexes mediate H2A ubiquitylation at polycomb target sites independently of PRC2 and H3K27me3. *Cell* **148**: 664-678.

van den Berg DL, Snoek T, Mullin NP, Yates A, Bezstarosti K, Demmers J, Chambers I, Poot RA. 2010. An Oct4-centered protein interaction network in embryonic stem cells. *Cell Stem Cell* **6**: 369-381.

Wang W, Lin C, Lu D, Ning Z, Cox T, Melvin D, Wang X, Bradley A, Liu P. 2008. Chromosomal transposition of PiggyBac in mouse embryonic stem cells. *PNAS USA* **105**: 9290-9295.
